# The Interaction between Collagen 1 and High Mannose Type CD133 Up‐Regulates Glutamine Transporter SLC1A5 to Promote the Tumorigenesis of Glioblastoma Stem Cells

**DOI:** 10.1002/advs.202306715

**Published:** 2023-11-23

**Authors:** Yuanyan Wei, Shuting Geng, Yu Si, Yuerong Yang, Qihang Chen, Sijing Huang, Xiaoning Chen, Wenlong Xu, Yinchao Liu, Jianhai Jiang

**Affiliations:** ^1^ NHC Key Laboratory of Glycoconjuates Research Department of Biochemistry and Molecular Biology School of Basic Medical Sciences Fudan University Shanghai 200032 P. R. China; ^2^ Division of Neurosurgery Zhongshan Hospital Fudan University Shanghai 200032 P. R. China; ^3^ Department of Neurosurgery Provincial Hospital Affiliated to Shandong First Medical University Jinan Shandong 250021 P. R. China

**Keywords:** CD133, COL1, glioma stem cell, SLC1A5, tumorigenesis

## Abstract

Targeting the niche components surrounding glioblastoma stem cells (GSCs) helps to develop more effective glioblastoma treatments. However, the mechanisms underlying the crosstalk between GSCs and microenvironment remain largely unknown. Clarifying the extracellular molecules binding to GSCs marker CD133 helps to elucidate the mechanism of the communication between GSCs and the microenvironment. Here, it is found that the extracellular domain of high mannose type CD133 physically interacts with Collagen 1 (COL1) in GSCs. COL1, mainly secreted by cancer‐associated fibroblasts, is a niche component for GSCs. COL1 enhances the interaction between CD133 and p85 and activates Akt phosphorylation. Activation of Akt pathway increases transcription factor ATF4 protein level, subsequently enhances SLC1A5‐dependent glutamine uptake and glutathione synthesis. The inhibition of CD133‐COL1 interaction or down‐regulation of SLC1A5 reduces COL1‐accelerated GSCs self‐renewal and tumorigenesis. Analysis of glioma samples reveals that the level of COL1 is correlated with histopathological grade of glioma and the expression of SLC1A5. Collectively, COL1, a niche component for GSCs, enhances the tumorigenesis of GSCs partially through CD133‐Akt‐SLC1A5 signaling axis, providing a new mechanism underlying the cross‐talk between GSCs and extracellular matrix (ECM) microenvironment.

## Graphical abstract

This study identifies COL1 as an extracellular binding molecule of cancer stem cells marker CD133. The interaction between COL1 and CD133 enhances the interaction between CD133 and p85, up‐regulates SLC1A5‐dependent glutamine uptake, and promotes the tumorigenesis of GSCs. This finding provides a new mechanism underlying the cross‐talk between GSCs and ECM microenvironment.

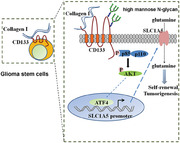

## 1 Introduction

Malignant astrocytic gliomas such as glioblastoma (GBM) are the most common and lethal intracranial tumors.^[^ ^1^ ^]^ A better understanding of the signaling molecules involved in GBM development helps to design efficient treatment of GBM.^[^ ^2^ ^]^ Cancer stem cells (CSCs) are responsible for tumor initiation, progression, and therapy resistance.^[^ ^3^ ^]^ For example, glioblastoma stem cells (GSCs) highly expressed stemness genes, displayed multi‐lineage differentiation capabilities, and were highly tumorigenic in immunocompromised mice.^[^ ^4^ ^]^ Exploring the mechanisms of high tumorigenic ability of CSCs, will help to develop new approaches for cancer therapy. Like normal stem cells, CSCs exist in a cellular niche comprised of numerous cell types.^[^ ^5^ ^]^ Targeting the niche components surrounding CSCs helps to develop more effective cancer treatments.^[^ ^6^ ^]^ However, the mechanisms underlying the crosstalk between CSCs and their surrounding microenvironment remain largely unknown.

CD133, a transmembrane glycoprotein, is widely used as a marker to isolate CSCs in several types of human tumors, including GBM.^[^ ^7^ ^]^ CD133 plays critical roles in tumor initiation, invasion, and therapy resistance.^[^ ^8^ ^]^ For example, we have reported that CD133 promotes the self‐renewal and tumorigenesis of GSCs through interaction with the PI3K regulatory subunit p85.^[^ ^8b^ ^]^ The interaction between CD133 and DNMT1 maintains the self‐renewal capacity of GSCs.^[^ ^9^ ^]^ CD133 promotes colon cancer cells proliferation through activating β‐catenin signaling.^[^ ^8a^ ^]^ Combined with that changing the sequence of CD133 extracellular region affects the binding of CD133 to intracellular molecules,^[^ ^10^ ^]^ clarifying the extracellular molecules binding to CD133 helps to elucidate the mechanism of the communication between CSCs and the microenvironment.

Accumulated evidence has shown that high expression of CD133 is associated with poor prognosis in several different types, including brain tumors, colorectal carcinoma, hepatoma, gastric carcinoma, and medulloblastoma.^[^ ^11^ ^]^ Anti‐CD133 antibody‐conjugated immunotoxins inhibited the progression of cancer.^[^ ^12^ ^]^ AC133‐specific CAR‐T cells recognized and killed patient‐derived GBM stem cells.^[^ ^13^ ^]^ Thus, CD133 is considered as a new target for cancer therapy. Elucidating the extracellular molecules binding to CD133 also helps to provide precise targets to block the tumor‐promoting function of CD133.

Here, we found that CD133 physically interacted with COL1 through its extracellular domain. COL1 enhanced the interaction between CD133 and p85 and subsequently promoted the self‐renewal and tumorigenesis of GSCs. Our findings illuminate the significance of the CD133‐COL1 interaction in the extracellular matrix microenvironment of GSCs.

## 2 Results

### 2.1 Collagen 1 is the Extracellular Binding Molecule of CD133

**Figure 1:**
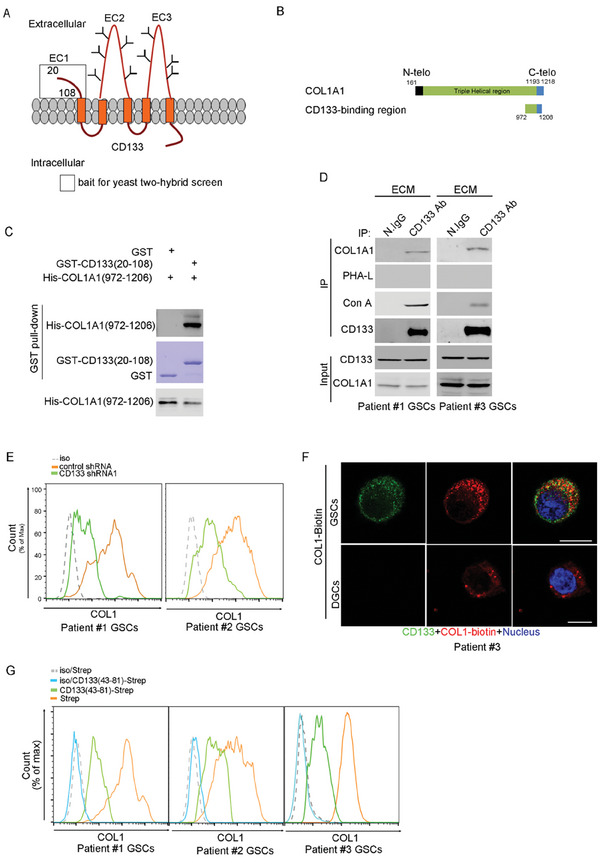
Extracellular COL1 binds to CD133 in GSCs. A) Graphic representation of the proposed structural model of CD133. This protein is modeled as having an extracellular N terminus, a cytoplasmic C terminus, two small cytoplasmic loops, and two large extracellular loops with nine *N*‐glycosylation site. N‐terminal extracellular domain of CD133 (residues 20–108) (indicated by square frame) was used as the bait for yeast two‐hybrid screen. B) Schematic of the COL1A1 molecule in mature COL1 consisting of the *N*‐telopeptide (*N*‐telo), the triple helical region, the *C*‐telopeptide (*C*‐telo) (aa 1193–1218). Yeast two‐hybrid system showed that CD133 interacted with the COL1 (aa 972–1206). C) In vitro interaction between CD133 and COL1. GST or GST‐CD133(20‐108) proteins were incubated with purified His‐COL1A1 (972‐1206) protein. The GST pull‐down products were blotted with anti‐His antibody. D) CD133 interacted with COL1 in vivo. The lysates of GSCs pre‐treated with ECM were subjected to IP using anti‐CD133 (Clone W6B3C1) antibody, followed by immunoblotting (IB) with anti‐CD133 antibody, anti‐COL1A1 antibody, anti‐PHA L lectin, and anti‐Con A lectin. Whole‐cell lysates were analyzed by IB with anti‐CD133 or anti‐COL1A1 antibodies as input. E) FACS analysis of the binding of COL1 to patient #1 (left panel) and patient #2 (right panel) GSCs expressing control shRNA (orange) or CD133 shRNA1 (green). F) GSCs and DGCs were incubated with Biotin‐COL1 at 4 °C, followed by staining with CD133 (green) and Biotin‐COL1 (red) to examine the interaction between exogenous COL1 and CD133. Scale bar, 10 µm. G) FACS analysis of the binding of COL I to patients #1–#3 GSCs pre‐treated with Strep or CD133(43‐81)‐Strep.

The yeast two‐hybrid screen was performed to identify proteins that bound to CD133 extracellular domain. CD133 contains an extracellular N‐terminal domain (EC1), two large extracellular loops (EC2 and EC3), and an intracellular C‐terminal domain (IC3).^[^ ^14^ ^]^ CD133 contains nine *N*‐glycosylation sites at the two large extracellular loops.^[^ ^15^ ^]^ Considering that *N*‐glycosylation in yeast was different from that in human,^[^ ^16^ ^]^ the extracellular N‐terminal domain of CD133 (residues 20–108) was used as the bait for the yeast two‐hybrid screen (**Figure** **1**A). The cDNA encoding extracellular N‐terminal domain of CD133 (residues 20–108) were cloned into pGBKT7 vector and was used as the bait to screen pACT2‐human cDNA libraries (human fetal brain). We isolated four positive clones from 1 × 10^6^ clones of a human fetal brain library. Among the positive clones, we identified one encoding partial sequence of COL1A1 (972‐1206), which was the only extracellular protein among the positive clones (Table S1, Supporting Information). COL1A1 encodes the α subunit of the type I collagen (COL1**),** which consists of the *N*‐telopeptide (*N*‐telo), the triple helical region, the *C*‐telopeptide (*C*‐telo) (Figure 1B).^[^ ^17^ ^]^ Extracellular N‐terminal domain of CD133 (residues 20–108) directly interacted with COL1A1 (aa 972–1206), as determined by GST pull‐down assay (Figure 1C). To validate the interaction between CD133 and COL1 in vivo, GSCs and their differentiated glioblastoma cell (DGC) were isolated from human GBM samples (Patient #1‐#3; pathological data see Table S2, Supporting Information). GSCs showed characteristics consistent with cancer stem cells: namely, neurosphere formation (Figure S1A, Supporting Information); expression of stem cell markers Sox2 and OLIG2 (Figure S1B,D, Supporting Information) and multilineage differentiation with markers for astrocytes (GFAP), neurons (MAP2) or oligodendrocytes (O4) (Figure S1E, Supporting Information). GSCs were highly tumorigenic in the brains of immunocompromised mice, and DGCs did not form detectable tumor (Figure S1F, Supporting Information). IP analysis of the lysis of GSCs co‐cultured with GBM tissues‐derived ECM showed that endogenous CD133 interacted with endogenous COL1 (Figure 1D). CD133 immunoprecipitated from GSCs was recognized by Con A lectin (recognizing high mannose), but not by PHA‐L lectin (recognizing β−1,6 branched *N*‐acetylglucosamine) (Figure 1D), indicating that the *N*‐glycan structure of CD133 was mainly high mannose type in GSCs. By FACS analysis, CD133 knockdown reduced the binding of COL1 to GSCs (Figure 1E). By immunofluorescence assay, COL1 bound to CD133 in GSCs (Figure 1F).

Next, we searched the region responsible for the interaction between CD133 and COL1. By GST pull‐down assay, deletion of COL1 amino acids 1193–1206 reduced the interaction between CD133 and COL1 in vitro (Figure S1G, Supporting Information), and CD133 N‐terminal segment (amino acids 43–81) interacted with COL1 in vitro (Figure S1H, Supporting Information). Strep pull‐down assay further confirmed that CD133 N‐terminal segment (amino acids 43–81) interacted with COL1 in vitro (Figure S1I, Supporting Information). Member protein extracellular domain is widely used to inhibit its interaction with its ligand.^[^ ^18^ ^]^ CD133(43‐81)‐Strep reduced the binding of COL1 to CD133, as determined by IP assay (Figure S1J, Supporting Information). By FACS assay, the binding of COL1 to the GSCs was significantly blocked by CD133(43‐81)‐Strep (Figure 1G). Taken together, COL1 is the extracellular binding molecule of CD133 in GSCs.

### 2.2 COL1 is a Niche Component for GSCs

**Figure 2:**
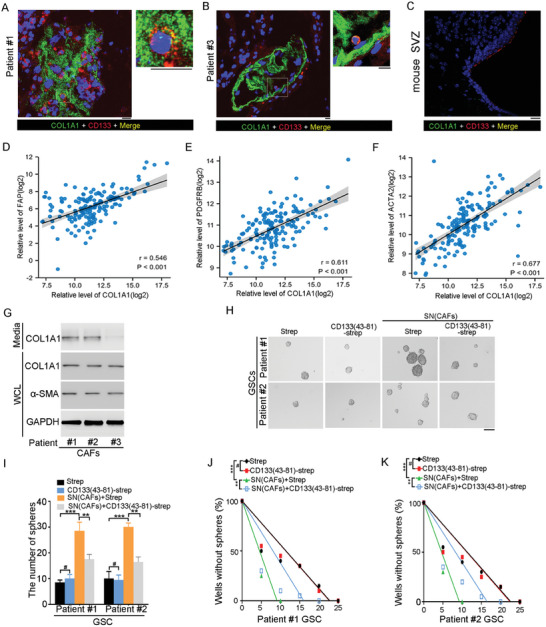
COL1 is a potential niche component for GSCs. A,B) The co‐localization of CD133 and COL1 was assessed by immunofluorescence staining of CD133 (red) and COL1 (green) in patient #1 (A) and #3 (B) glioblastoma tissues. Nucleus (blue) were counterstained with DAPI. Co‐localization of CD133 and COL1 is demonstrated by yellow fluorescence. The high‐power image of the framed regions in the left panels is showed in the right panels. Scale bars, 10 µm. C) Immunofluorescence analysis of CD133 (red) and COL1A1 (green) in the SVZ area of mouse. Scale bars, 10 µm. D–F) The correlation between COL1A1 expression with fibroblast marker markers FAP (D), PDGFRB (E), and ACTA2 (F) in GBM tissues by TCGA database analysis was indicated. Numbers represent coefficient values (*n* = 160). G) Western blot analysis of COL1A1 expression in conditional media from fibroblasts isolated from patients #1–#3 GSCs. H,I) Single cell sphere formation assay of GSCs treated with the supernatant from CAFs and Strep or CD133(43‐81)‐Strep. H) Representative images of sphere are shown. Scale bar, 10 µm. I) Results are expressed as mean ± SD (*n* = 3; **p < 0.01, ***p < 0.001, #, ns). J,K) Limiting dilution assay analyses the frequency in GSCs isolated from Patient #1 (J) and Patient #2 (K) treated with supernatant from CAFs and Strep or CD133(43‐81)‐Strep. *n* = 10, ***p < 0.001 by ELDA analysis.

GSCs reside in GSC niches, including perivascular niches and necrosis niches.^[^ ^19^ ^]^ Supporting this notion, by IHC analysis of stem cell marker CD133 in GBM tissues. GSCs were located in vascular niche (Figure S2A, Supporting Information), and necrosis area (Figure S2B, Supporting Information). By immunofluorescence assay, the juxtaposition of GSCs with COL1 was detected in GBM tissues and COL1 was co‐localized with CD133 (**Figure** **2**A,B). Normal neural stem cells reside in the subventricular zone (SVZ) of the adult mammalian brain. By immunofluorescence assay, the level of COL1 was low in the SVZ of the adult mammalian brain in mice. The co‐localization between COL1 and CD133 was not observed in the SVZ of the adult mice brain (Figure 2C). Thus, COL1 is a niche component for GSCs.

COL1 was mainly secreted by cancer‐associated fibroblasts in various kinds of cancer.^[^ ^20^ ^]^ TCGA data analysis also showed that the expression of COL1A1 was positively correlated with the markers of fibroblasts including FAP, PDGFRB, ACTA2, S100A4, and VIM in GBM tissues (Figure 2D–F; Figure S2C,D, Supporting Information). By western blot analysis of condition media, fibroblasts isolated from GBM tissues secreted COL1 (Figure 2G). However, GSCs have low expression of COL1 as compared to fibroblasts (Figure S2E, Supporting Information). Thus, fibroblasts were the major cells responsible for the secretion of COL1 in GBM tissues.

To examine the contribution of COL1 in fibroblasts‐regulated GSCs self‐renewal, cancer‐associated fibroblast were transduced with shRNA targeting COL1. Knockdown of COL1 reduced the positive effect of conditional medium of CAFs on the sphere formation of GSCs (Figure S2F,G, Supporting Information). However, the conditional medium of CAFs increased the number of sphere formed by GSCs, which was blocked by CD133(43‐81)‐Strep (Figure 2H,I). Supporting this notion, conditional medium of CAFs enhanced the sphere‐forming frequency of GSCs by limited dilution assay, which was inhibited by CD133(43‐81)‐Strep (Figure 2J,K). Together, COL1 secreted from CAFs promotes the self‐renewal of GSCs partially through interaction with CD133.

### 2.3 The Interaction between COL1 and CD133 Enhances the Self‐Renewal and Tumorigenic Capacities of GSCs

**Figure 3:**
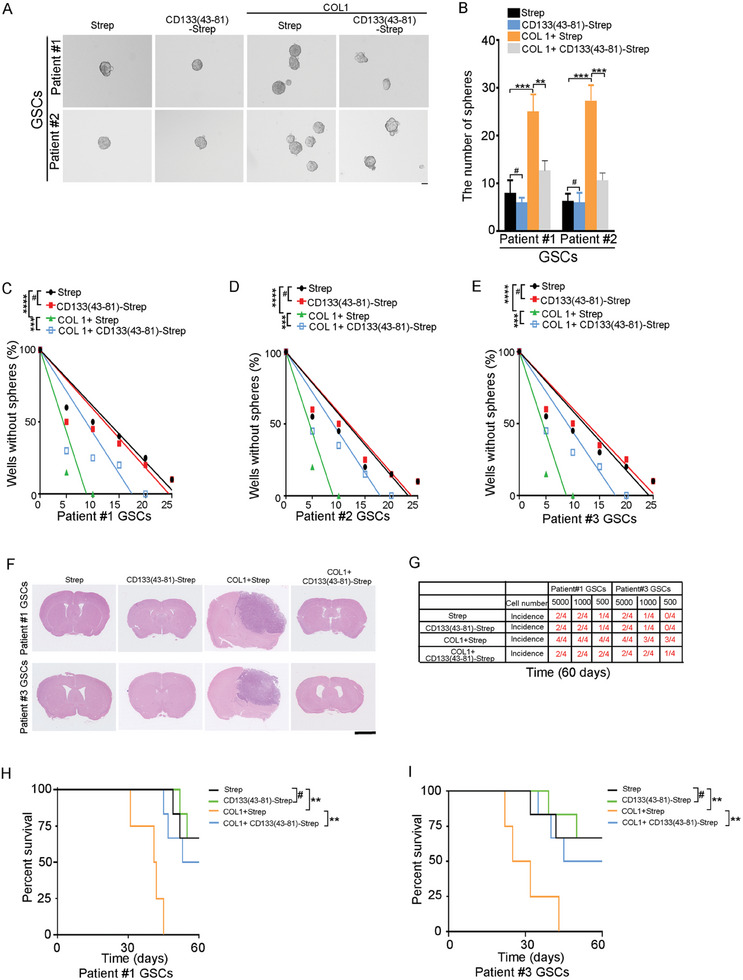
The interaction between COL1 and CD133 promotes the self‐renewal and tumorigenesis of GSCs. A,B) Single cell sphere formation assay of GSCs treated with Strep or CD133(43‐81)‐Strep and control or COL1 (10 µg ml^−1^). A) Representative images of sphere are shown. Scale bar, 10 µm. B) Results are expressed as mean ± SD (*n* = 3; **p < 0.01, ***p < 0.001, #, ns). C–E) Limiting dilution assay shows that the COL1‐CD133 interaction increased stem cell frequency in Patients #1–#3 GSCs (C‐E). *n* = 10, ***p < 0.001 by ELDA analysis. F,G) The tumor‐initiating capacity of GSCs treated with Strep or CD133(43‐81)‐Strep and control or COL1. An intracranial limiting dilution tumor formation assay (employing 5000, 1000, and 500 cells per mouse) was performed using GSCs treated with Strep or CD133(43‐81)‐Strep and control or COL1. F) H&E staining of mouse brain shows tumors formation by GSCs treated with Strep or CD133(43‐81)‐Strep and control or COL 1. Scale bar, 2.5 mm. G) The table displays the number of mice developing tumor. H,I) Patient #1 GSCs (H) and Patient #3 GSCs (I) were intracranially co‐implanted with Strep or CD133(43‐81)‐Strep and control or COL1 into the brain of immunocompromised mice (5,000 cells per mouse). Mice were sacrificed when they were moribund or 60 days after implantation. Survival of mice (*n* = 6) was evaluated by Kaplan–Meier analysis (**p < 0.01, log rank test).

We next evaluated the role of COL1‐CD133 interaction in the self‐renewal and tumorigenesis of GSC. The neurosphere formation assay is a conventional method to measure the self‐renewal capacity of GSC.^[^ ^21^ ^]^ COL1 increased the number of neurosphere formed by GSCs. The effect of COL1 on sphere formation of GSCs was blocked by CD133(43‐81)‐Strep (**Figure** **3**A,B) or CD133 knockdown (Figure S3A,B, Supporting Information). Limiting dilution analysis is a method to estimate the activity of stem cells.^[^ ^22^ ^]^ COL1 increased the sphere‐forming frequency of GSCs by limited dilution assay. The effect of COL1 on the sphere‐forming frequency of GSCs was blocked by CD133(43‐81)‐Strep or CD133 knockdown (Figure 3C–E; Figure S3C,D, Supporting Information). Thus, the interaction between COL1 and CD133 enhances the self‐renewal capacity of GSCs.

Next, to examine the effect of COL1‐CD133 interaction on the tumor‐initiating capacity of GSCs, GSCs either alone or in combination with COL1 and Strep or CD133(43‐81)‐Strep were implanted into the brains of immunocompromised mice. After injection into the mice for 60 days, GSCs alone showed low frequency of tumor formation. However, mixed COL1 and GSCs promoted tumor formation (Figure 3F,G). The effect of COL1 on the tumor formation of GSCs was blocked by CD133(43‐81)‐Strep (Figure 3F,G). Accordingly, co‐implantation of COL1 reduced the survival of xenograft‐bearing mice compared to GSCs alone, which could be partially rescued by CD133(43‐81)‐Strep (Figure 3H,I). Together, COL1 promotes the self‐renewal and tumorigenesis of GSCs partly through interaction with CD133.

### 2.4 The COL1‐CD133 Interaction Activates the PI3K‐Akt Pathway in GSCs

**Figure 4:**
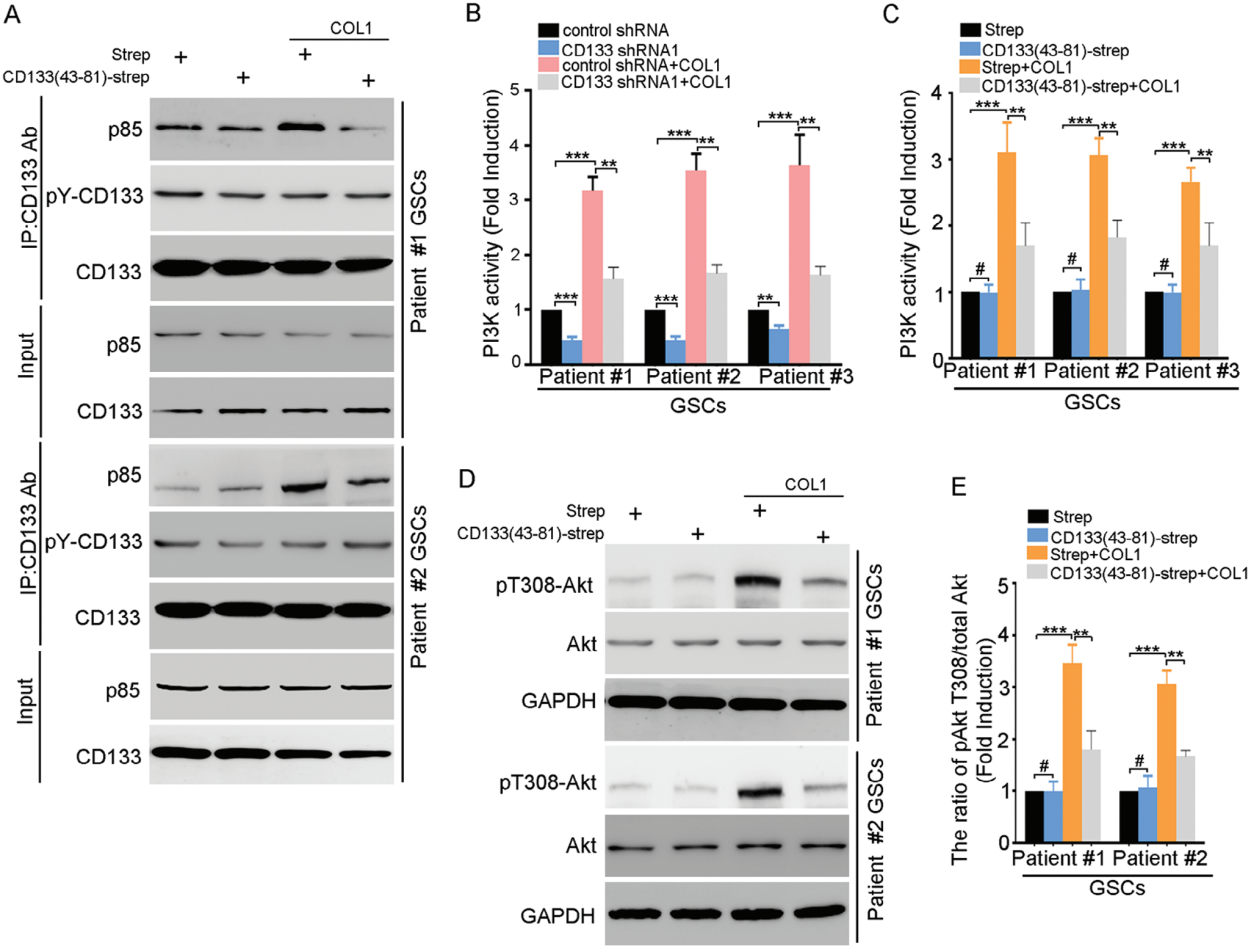
The interaction between COL1 and CD133 activates PI3K‐Akt pathway. A) Co‐immunoprecipitation (Co‐IP) analysis to determine the effect of COL1 on the interaction between CD133 and p85 in vivo. The lysates of GSCs pre‐treated with COL I and Strep or CD133(43‐81)‐Strep were subjected to IP using anti‐CD133 or anti‐p85 antibody, followed by IB (immunoblotting) with anti‐CD133, anti‐p85 or anti‐pY antibodies. Whole‐cell lysates were analyzed by IB with anti‐CD133 or anti‐p85 antibodies as Input. B) The PI3K activity of GSCs expressing control shRNA or CD133 shRNA1 were assessed pre‐treated with COL I and Strep or CD133(43‐81)‐Strep for 1 h were assessed using a PI3 kinase ELISA kit. Values are normalized to that of GSCs. Results are expressed as mean ± SD (*n* = 3; ***p < 0.001, **p < 0.01) C) The PI3K activity of GSCs pre‐treated with COL1 and Strep or CD133(43‐81)‐Strep were assessed using a PI3 kinase ELISA kit. Values are normalized to that of GSCs. Results are expressed as mean ± SD (*n* = 3; ***p < 0.001, **p < 0.01, #,ns). D,E) The activity of Akt signaling in GSCs pre‐treated with COL I and Strep or CD133(43‐81)‐Strep. Whole‐cell lysates were analyzed by western blotting. GAPDH was blotted as a loading control. D) The representative figures are presented out of three separate experiments. E) The relative densities of pT308‐Akt (D) to total Akt in (D) were quantified using densitometry. Values are normalized to that of GSCs treated with control. Results are expressed as mean ± SD (*n* = 3; ***p < 0.001, **p < 0.01).

Next, we investigated the mechanisms by which the COL1‐CD133 interaction promoted the self‐renewal of GSCs. The interaction between CD133 and p85 activates PI3K‐Akt pathway.^[^ ^8^, ^10^ ^]^ COL1 increased the CD133‐p85 binding without obviously changing the level of CD133 phosphorylation, which was blocked by CD133(43‐81)‐Strep (**Figure** **4**A). CD133 N‐terminal domain (aa20‐108) binds to GM1 (ganglioside M1).^[^ ^23^ ^]^ Mutations in the GM1‐binding site of CD133 stimulate its interaction with p85.^[^ ^10^ ^]^ GM1‐binding ELISA assay showed that COL1 inhibited the binding of CD133(20‐108) to GM1 (Figure S4A, Supporting Information).

Considering that PI3K activates Akt signaling depending on its lipid kinase activity,^[^ ^24^ ^]^ the effect of COL1‐CD133 interaction on PI3K activity was evaluated by PI3K activity ELISA kit. COL1 increased the activity of PI3K, and down‐regulation of CD133 reduced COL1‐induced PI3K activity (Figure 4B). And, the effect of COL1 on the PI3K activity was blocked by CD133(43‐81)‐Strep (Figure 4C). Akt achieves its full activity through phosphorylation at both threonine 308 (T308) and serine 473 (S473).^[^ ^25^ ^]^ COL1 increased the level of the phosphorylation of Akt at T308. However, the effect of COL1 on the Akt phosphorylation was reduced by CD133(43‐81)‐Strep (Figure 4D,E), or CD133 down‐regulation (Figure S4B, Supporting Information). Mdm2 and FOXO are target for phosphorylation by Akt.^[^ ^26^ ^]^ COL1 increased the level of the phosphorylation of MDM2 and FOXO1, which was reduced by CD133(43‐81)‐Strep (Figure S4C,D, Supporting Information). Collectively, the interaction between COL1 and CD133 activates PI3K‐Akt pathway in GSCs.

### 2.5 Activation of PI3K‐Akt Pathway by the COL1‐CD133 Interaction Enhances the Glutamine Uptake Through Up‐Regulating SLC1A5

**Figure 5:**
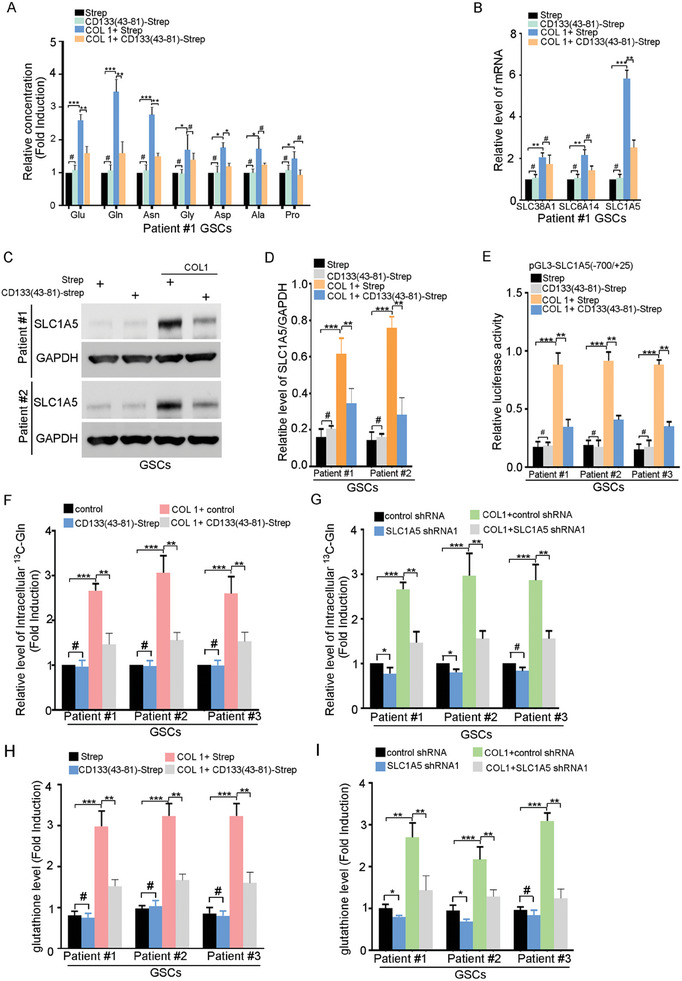
The interaction between COL1 and CD133 up‐regulates SLC1A5 in GSCs. A) The intracellular amino acid concentration in GSCs treated with Strep or CD133(43‐81)‐Strep and control or COL I was measured by mass spectrometry. Results are expressed as mean ± SD (*n* = 3; ***p < 0.001, **p < 0.01, *p < 0.05, #, ns). B) The mRNA level of glutamine transporter including SLC38A1, SLC6A14, and SLC1A5 in GSCs treated with Strep or CD133(43‐81)‐Strep and control or COL I (10 µg ml^−1^) was examined by qRT‐PCR. Results are expressed as mean ± SD (*n* = 3; ***p < 0.001). C,D) Western blot analysis of SLC1A5 expression in GSCs treated with control or COL I or/and Strep or CD133(43‐81)‐Strep as examined by western blot. C) Representative image was shown. D) Results are expressed as mean ± SD (*n* = 3; ***p < 0.001, **p < 0.01, *p < 0.05, #, ns) E) GSCs transiently transfected with pGL3‐SLC1A5(−700/+25) and pRL were treated with Strep or CD133(43‐81)‐Strep and control or COL I (10 µg ml^−1^). Seventy‐two hours later, the luciferase activity was normalized to Renilla luciferase activity. Results are expressed as mean ± SD (*n* = 3; **p < 0.01, ***p < 0.001). F) GSCs pretreated with control or COL I or/and Strep or CD133(43‐81)‐Strep were incubated with 13*C‐L*‐glutamine to assess *L*‐glutamine uptake. Values are normalized to that of GSCs treated with control. Results are expressed as mean ± SD (*n* = 3; ***p < 0.001,**p < 0.01, *p < 0.05, #, ns) G) GSCs expressing control shRNA or SLC1A5 shRNA1 pretreated with control or COL1 were incubated with 13*C‐L‐*glutamine to assess *L‐*glutamine uptake. Values are normalized to that of GSCs treated with control. Results are expressed as mean ± SD (*n* = 3; ***p < 0.001, **p < 0.01, *p < 0.05, #, ns) H) The intracellular glutathione levels in GSCs with Strep or CD133(43‐81)‐Strep and control or COL1 were assessed. Values are normalized to that of GSCs treated with control. Results are expressed as mean ± SD (*n* = 3; ***p < 0.001). I) The intracellular glutathione levels in GSCs expressing control shRNA or SLC1A5 shRNA1 pretreated with control or COL1 were assessed with glutathione colorimetric assay kit. Values are normalized to that of GSCs treated with control. Results are expressed as mean ± SD (*n* = 3; ***p < 0.001).

The uptake of amino acids regulates the self‐renewal and tumorigenesis of cancer stem cells.^[^ ^27^ ^]^ Using mass spectrometry, COL1 significantly increased the intracellular abundance of glutamine and glutamate, which could be blocked by the addition of CD133(43‐81)‐Strep (**Figure** **5**A). Glutamine transporter mainly includes SLC38A1, SLC1A5 and SLC6A14.^[^ ^28^ ^]^ COL1 increased the level of SLC1A5 mRNA expression, without significantly changing the expression of SLC38A1 and SLC6A14 in GSCs (Figure 5B). The effect of COL1 on the SLC1A5 protein expression and mRNA expression was blocked by CD133(43‐81)‐Strep or CD133 knockdown (Figure 5C,D; Figure S5A, Supporting Information). The interaction between CD133 and p85 activates PI3K‐Akt pathway.^[^ ^8b^ ^]^ The positive effect of COL1 on SLC1A5 expression was significantly blocked by PI3K pathway inhibitor Wortmannin (Figure S5B, Supporting Information). Thus, activation of PI3K‐Akt pathway by the COL1‐CD133 interaction up‐regulates SLC1A5.

To clarify the mechanism of for Akt‐induced SLC1A5 expression, we generated a series of constructs containing the fragments of the human SLC1A5 promoter. By dual luciferase assay, a deletion from −700 to −447 resulted in a decrease in the promoter activity and mostly loss of CA‐Akt activation (Figure S5C, Supporting Information). ATF4 binds to the ATF binding site between −700 and −447 in human SLC1A5 promoter.^[^ ^29^ ^]^ Supporting this notion, chromatin immunoprecipitation (ChIP) analysis showed the binding of ATF4 to SLC1A5 promoter in COL1‐treated GSCs (Figure S5D, Supporting Information). Western blot analysis showed that CA‐Akt increased the level of ATF4 protein expression (Figure S5E, Supporting Information). Down‐regulation of ATF4 by ATF4 RNA interference blocked up‐regulation of SLC1A5 promoter by CA‐Akt (Figure S5F, Supporting Information). Thus, Akt pathway promotes SLC1A5 expression partly through up‐regulating ATF4. Furthermore, the effect of COL1 on the activity of SLC1A5 promoter was blocked by Wortammin (Figure S5G, Supporting Information), or CD133(43‐81)‐strep (Figure 5E). These finding indicated the COL1‐CD133 interaction enhances SLC1A5 transcription through the activation of PI3K‐Akt pathway.

SLC1A5 is a Na+‐dependent neutral amino acid exchanger that can transport glutamine, alanine, serine, asparagine, and threonine.^[^ ^28^, ^30^ ^]^ By ^13^C‐glutamine uptake assay, SLC1A5 down‐regulation reduced the uptake of glutamine in GSCs (Figure S5H, Supporting Information). COL1 promoted the uptake of glutamine in GSCs, which could be blocked by CD133(43‐81)‐Strep (Figure 5F), or SLC1A5 down‐regulation (Figure 5G). Gln participates in mitochondrial metabolism, synthesis of the cellular primary antioxidant glutathione, among other activities.^[^ ^31^ ^]^ Interestingly, the total level of intermediate metabolites of the TCA cycle, α‐ketoglutarate (α‐KG) and acetyl‐CoA, were not significantly changed in SLC1A5‐knockdown GSCs (Figure S5I,J, Supporting Information). Consistent with this, SLC1A5 down‐regulation did not obviously reduce the amount of the ^13^C labeling patterns of TCA intermediates in GSCs fed with ^13^C‐glutamine tracer (Figure S5K–M, Supporting Information). However, SLC1A5 knockdown reduced the level of glutathione (Figure S5N, Supporting Information). Furthermore, COL I increased the level of glutathione, which was blocked by SLC1A5 knockdown or CD133(43‐81)‐Strep (Figure 5H,I). Collectively, the interaction between COL1 and CD133 enhances the glutamine uptake in GSCs through up‐regulation of SLC1A5.

### 2.6 Down‐Regulation of SLC1A5 Partially Inhibited the Effect of COL1 on the Tumorigenesis of GSCs

We next evaluated the contribution of SLC1A5 in the effect of COL1‐CD133 interaction on the tumorigenesis of GSCs. First, SLC1A5 knockdown in GSCs by lentivirus‐based systems (Figure S6A, Supporting Information), decreased the spheres formation of patient #1 and #2 GSCs, but not of patient #3 GSCs (Figure S6B,C, Supporting Information). Treatment with *N*‐acetyl‐cysteine (NAC), a precursor to glutathione, but not a widely used cell‐permeable derivative of α‐KG dimethyl‐α‐KG, reversed the negative effect of SLC1A5 knock down on the self‐renewal capacity of GSCs (Figure S6D, Supporting Information). V‐9302 selectively and potently targets the amino acid transporter SLC1A5.^[^ ^32^ ^]^ V‐9302 inhibited the self‐renewal capacity of GSCs (Figure S6E, Supporting Information). Second, SLC1A5 overexpression increased the spheres formation of patient #1‐#3 GSCs (Figure S6F,G, Supporting Information). However, Buthionine sulphoximine (BSO), an inhibitor of gamma‐glutamylcysteine synthetase, effectively reduces glutathione synthesis. BSO significantly reduced SLC1A5‐induced glutathione (Figure S6H, Supporting Information), and inhibited SLC1A5‐induced neurospheres formation (Figure S6I, Supporting Information). Finally, SLC1A5 knockdown reduced the tumorigenic ability of GSCs (Figure S6J, Supporting Information). V9302 reduced the tumorigenesis of GSCs (Figure S6K, Supporting Information). SLC1A5 overexpression increased the tumorigenesis ability of GSCs (Figure S6L,M, Supporting Information). Together, up‐regulation of SLC1A5 enhances the self‐renewal and tumorigenic abilities of GSCs.

**Figure 6:**
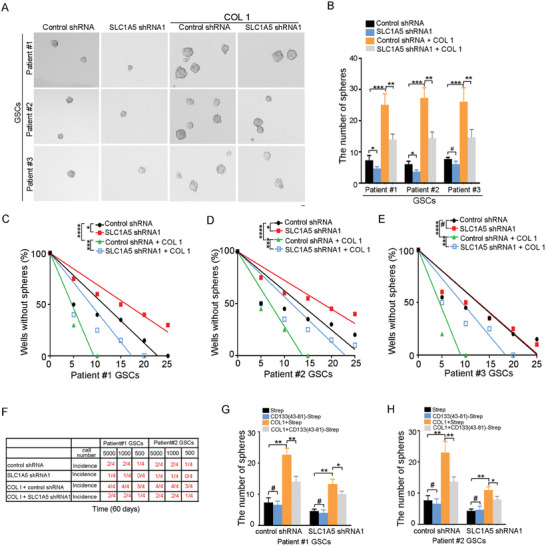
Down‐regulation of SLC1A5 partially inhibited the effect of COL1 on the tumorigenesis of GSCs. A,B) Single cell sphere formation assay of GSCs expressing control shRNA or SLC1A5 shRNA1 treated with COL1. A) Representative images of sphere are shown. Scale bar, 10 µm. B) Results are expressed as mean ± SD (*n* = 3; ***p < 0.001). C–E) Limiting dilution assay examined the stem cell frequency in patient #1 (C), patient #2 (D), or patient #3 (E) GSCs expressing control shRNA or SLC1A5 shRNA treated with COL1. *n* = 10, ***p < 0.001, **p < 0.01, *p < 0.05 by ELDA analysis. F) GSCs expressing control shRNA or SLC1A5 shRNA1 treated with COL1. An intracranial limiting dilution tumor formation assay (employing 5000, 1000, 500 cells per mouse) was performed using GSCs and control or COL1. The table displays the number of mice developing tumors. G,H) Single cell sphere formation assay of patient #1 (G) and patient #2 H) GSCs expressing control shRNA or SLC1A5 shRNA treated with Strep or CD133(43‐81)‐Strep and control or COL1. Results are expressed as mean ± SD (*n* = 3; **p < 0.01, *p < 0.05).

Next, the effects of SLC1A5 in COL1‐ehanced self‐renewal and tumorigenesis of GSCs were examined. First, down‐regulation of SLC1A5 partially reduced the neurosphere formation of GSCs enhanced by COL1 (**Figure** **6**A,B). Second, the effect of COL1 on the sphere‐forming frequency of GSCs could be partially reduced by SLC1A5 down‐regulation (Figure 6C–E). Third, to determine the impact of COL1 on the survival of tumor‐bearing mice, GSCs expressing control shRNA or SLC1A5 shRNA either alone or in combination with COL1 were implanted into the mice brains. SLC1A5 knockdown partially reduced the positive effect of COL1 on the tumorigenesis capacity of GCSs (Figure 6F). Together, the COL1 promotes the self‐renewal and tumorigenesis of GSCs at least partly through up‐regulation of SLC1A5.

Next, the effects of SLC1A5 in COL1‐CD133 interaction‐enhanced self‐renewal and tumorigenesis of GSCs were examined. The self‐renewal and tumorigenesis ability of GSCs expressing control shRNA or SLC1A5 shRNA treated with Strep or CD133(43‐81)‐Strep and control or COL1 were examined. The effect of COL1 on the sphere‐forming frequency of GSCs was significantly blocked by CD133(43‐81)‐Strep in GSCs expressing control shRNA, but not in GSCs expressing SLC1A5 shRNA (Figure 6G,H). Furthermore, the effect of COL1 on the tumorigenesis frequency of GSCs was significantly blocked by CD133(43‐81)‐Strep in GSCs expressing control shRNA, but not in GSCs expressing SLC1A5 shRNA (Figure S6N, Supporting Information). Collectively, the COL1‐CD133 interaction enhanced self‐renewal and tumorigenesis of GSCs at least partly through up‐regulating SLC1A5.

### 2.7 The Level of COL1 was Correlated with Glioma Grades and the Survival of Glioma Patient

**Figure 7:**
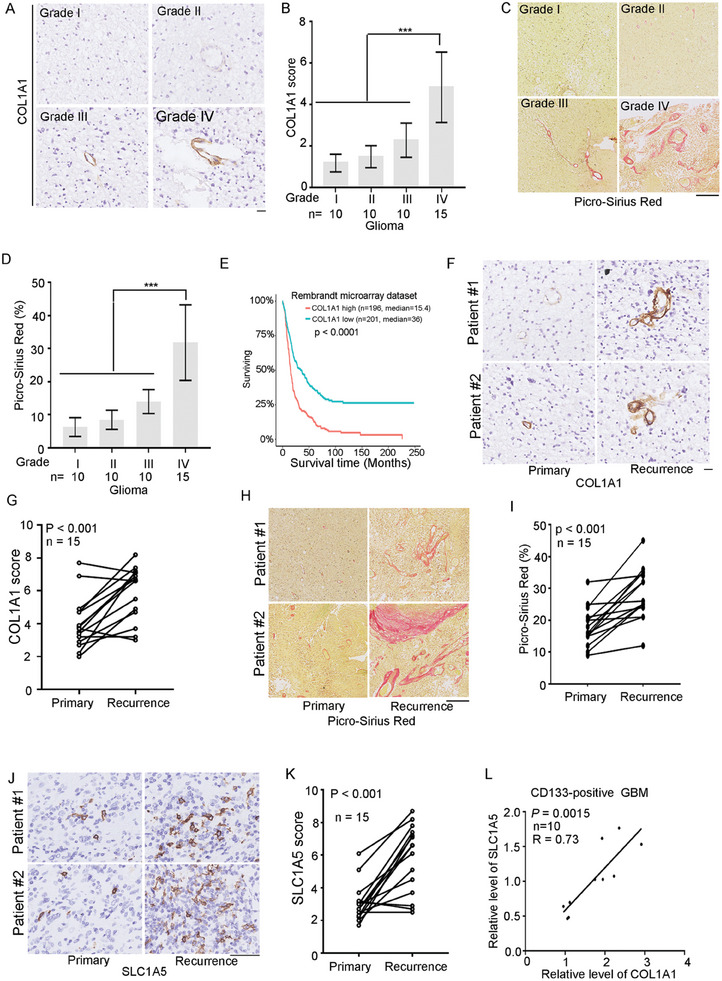
The level of COL1 was positively correlated with glioma grades and the level of SLC1A5. A,B) Expression of COL1 was examined by IHC staining in 45 gliomas samples of different grades. A) Representative microphotographs of immunohistochemical staining of COL1 in glioma grade I–IV tissues. B) The scores for quantitative staining of COL1 in the tissue sections were determined according to a total score (range, 0–8) that was obtained by combining the score of the percentage of positive cells and the score of the staining intensity. Values are mean ± SD. The level of COL1 in glioma with different grade was analyzed (Student's *t*‐test, two‐tailed, *p < 0.05, **p < 0.01, ***p < 0.001). Scale bar, 20 µm. C,D) Gliomas samples of different grades were stained with picrosirius red to visualize collagen. C) Representative images were shown. Scale bar, 200 µm. D) Picrosirius red quantification showed that the collagen content was significantly increased in IV gliomas when compared with grade I–III gliomas; ***p < 0.001. E) High expression of COL1A1 was related to worse overall outcome in glioma samples from Kaplan–Meier plotter in Rembrandt microarray. F,G) IHC analysis of COL1A1 in 15 paired primary and recurrent glioma sections. F) Representative microphotographs of immunohistochemical staining of COL1A1 in 15 paired primary and recurrent glioma sections. Scale bar represents 20 µm. G) The scores for quantitative staining of COL1A1 in the tissue sections were determined. *n* = 15, ***p < 0.001. Student's *t*‐test. H,I) Fifteen paired primary and recurrent glioma sections were stained with picrosirius red to visualize collagen. H) Representative images were shown. Scale bar, 200 µm. I) Picrosirius red quantification showed that the collagen content was significantly increased in recurrent gliomas when compared with primary gliomas; ***p < 0.001, *n* = 15. J,K) IHC analysis of SLC1A5 in 15 paired primary and recurrent glioma sections. J) Representative microphotographs of immunohistochemical staining of SLC1A5 in 15 paired primary and recurrent glioma sections. Scale bar represents 200 µm. K) The scores for quantitative staining of SLC1A5 in the tissue sections were determined. Values are mean ± SD (*n* = 15). ***p < 0.001. Student's *t*‐test. L) QRT‐PCR analysis of COL1A1 and SLC1A5 expression of GBM tissues expressing CD133. The correlation of COL1A1 expression with SLC1A5 expression in GBM tissues was analyzed using correlation analysis (*n* = 10).

To examine the expression of COL1 during malignant progression of human brain tumors, IHC staining was performed on paraffin‐embedded section from human glioma of different grades using the antibodies recognizing COL1. As shown in **Figure** **7**A, human grade I‐II gliomas showed negative‐to‐lower levels of COL1 and grade III glioma showed a moderate level of COL1. In contrast, grade IV glioma displayed a high level of COL1. Statistical analysis for the level of COL1 in a panel of gliomas samples showed high expression of COL1 in glioma grade IV tissues (Figure 7B). Accordingly, the level of COL1A1 mRNA was high in glioma grade IV tissues by TCGA glioma dataset analysis (Figure S7A, Supporting Information). Picrosirius red staining is a commonly used histological technique to visualize collagen in tissue sections.^[^ ^33^ ^]^ Picrosirius red staining showed that there was a marked increase of collagen deposition in grade IV glioma as compared to grade I‐III glioma (Figure 7C,D). More importantly, those glioma patients with high expression of COL1 had significantly poorer OS than those with lower expression in Rembrandt, Gravendeel and TCGA datasets (Figure 7E; Figure S7B,C, Supporting Information). Taken together, the level of COL1 was highly expressed in glioma IV grade tissues.

By immunohistochemical (IHC) staining on paraffin‐embedded sections from paired primary and recurrent tissues, the level of COL1 in recurrent tissues was significantly higher than in primary tissues (Figure 7F,G). Picrosirius red staining showed that there was a marked increase of collagen deposition in recurrent tissues was significantly higher than in primary tissues (Figure 7H,I). The level of SLC1A5 in recurrent tissues was significantly higher than in primary tissues (Figure 7J,K). Thus, the expression of COL1 was associated with glioma recurrence.

Considering our finding that the interaction between COL1 and CD133 upregulates SLC1A5, we predicted a significant association between COL1 and SLC1A5 expression in GBM samples. By immunofluorescence staining, co‐expression of CD133 and SLC1A5 was observed in GBM samples (Figure S7D, Supporting Information). By qRT‐PCR analysis, the level of COL1 mRNA expression was positive correlated with the level of SLC1A5 mRNA expression in CD133‐positive GBM samples (Figure 7L). The relationship between COL1A1 and glutamine metabolism‐related gene expression was further evaluated using TCGA data. TCGA data analysis also showed that the expression of COL1A1 was positively correlated with the expression of SLC1A5 in GBM tissues and negative correlated with the expression of GLUD1 and GLUD2 in GBM tissues (Figure S7E–L, Supporting Information).

## 3 Discussion

**Figure 8:**
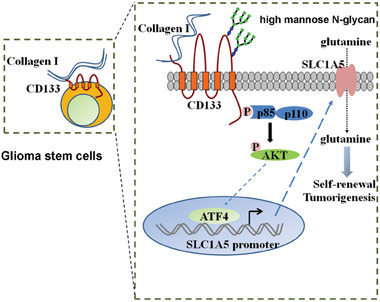
Model of the CD133‐COL1 interaction promoting the tumorigenesis of glioma stem cells.

CD133 is preferentially localized in plasma membrane protrusions such as microvilli and primary cilia in epithelial cells and stem cells,^[^ ^34^ ^]^ raising the possibility that CD133 regulates the communication between cells and the microenvironment. Here, we demonstrated that the interaction between COL1 and CD133 enhanced the interaction between CD133 and p85 and increased Akt phosphorylation level. Activation of Akt pathway enhanced the expression of ATF4, resulting in up‐regulation of ATF4 target gene SLC1A5. The COL1‐CD133 interaction promoted the tumorigenesis of GSCs partly through up‐regulation of SLC1A5‐dependent glutamine uptake (**Figure** **8**). Collectively, these data demonstrate that the interaction between COL1 and CD133 enhances glutamine uptake to promote the tumorigenesis of GSCs, providing a new mechanism underlying the cross‐talk between GSCs and ECM microenvironment.

Increasing evidence suggests that the ECM serves as a niche for CSCs.^[^ ^35^ ^]^ COL1, an important part of the extracellular matrix, has been linked to tumor growth and metastasis.^[^ ^36^ ^]^ Furthermore, COL1 plays an important role as a scaffold of the CD133‐positive GSCs.^[^ ^37^ ^]^ Supporting this notion, COL I promotes the expansion of CSCs.^[^ ^38^ ^]^ Our findings illuminate the significance and mechanism of the interaction between CD133 and COL1 in the extracellular matrix microenvironment of GSCs. Considering that CD133 is the marker of glioma stem cells, our finding provides a new signal pathway medicating the crosstalk between GSCs and ECM microenvironment. However, CD133 ectodomain (aa 43–81) as well as by CD133 gene silencing does not completely inhibit COL1‐accelerated GSCs self‐renewal and tumorigenesis. A plausible explanation for this phenomenon is the presence of other receptors for COL I in GSCs. DDR1, DDR2, and partial members of the integrin family (including α1β1, α2β1, α10β1, and α11β1 function as collagen receptors.^[^ ^39^ ^]^ COL1 enhances the interaction between CD133 and p85 without changing the phosphorylation of CD133. Mutations in the GM1‐binding site of CD133 stimulate its interaction with p85.^[^ ^10^ ^]^ COL1 inhibited the binding of CD133 N‐terminal to GM1. Thus, we speculate that COL1 enhances the interaction between CD133 and p85 partly through inhibiting the binding of CD133 to GM1. However, the contribution of GM1 in the COL1‐CD133 interaction regulating CD133‐p85 interaction needs further examination.

Another finding is that the interaction between COL1 and CD133 promotes glutamine uptake through up‐regulation of glutamine transporter SLC1A5. Glutamine is physiologically an essential source of carbon and nitrogen for cancer cell proliferation.^[^ ^40^ ^]^ SLC1A5‐dependent glutamine uptake is an essential role for the self‐renewal of cancer stem cells.^[^ ^41^ ^]^ However, the contribution of SLC1A5 in GSCs self‐renewal and tumorigenesis remains elusive. We provided evidence that up‐regulation of SLC1A5 enhanced the self‐renewal of GSCs. However, down‐regulation of endogenous SLC1A5 partially inhibited the self‐renewal ability of patient #1 and patient #2 GSCs, but not obviously reduced the self‐renewal ability of patient #3 GSCs. A plausible explanation for this phenomenon is that SLC1A5 is lowly expressed in partially CD133+ glioma cells isolated from GBM tissues.^[^ ^42^ ^]^ Another explanation for this phenomenon is that glutamine transporter mainly includes SLC38A1, SLC1A5, and SLC6A14.^[^ ^43^ ^]^ SLC1A5 knockdown reduced the level of glutathione. Glutathione depletion with BSO blocked the effect of SLC1A5 on the self‐renewal of glioma stem cells. Glutathione is essential for self‐renewal of cancer stem cells.^[^ ^44^ ^]^ Thus, we speculate that SLC1A5 regulates the self‐renewal of GSCs partially through up‐regulation of glutathione.

Several studies have proved that attached stem cells or attached tumor cells go through a metabolic reprogramming in which glutamine is less utilized.^[^ ^45^ ^]^ However, our finding showed that COL1 enhances glutamine uptake in GSCs. A plausible explanation for this phenomenon is that the relationship between COL1 and the utilization of glutamine by tumor cells is related to the concentration of collagen. Under high density of Collagen I conditions, the contribution of glutamine as a fuel source to drive the TCA cycle was significantly enhanced.^[^ ^46^ ^]^ The level of COL1 was high in GBM tissues.^[^ ^47^ ^]^ Second, The ECM mainly contained collagen fibers. Our results showed that the molecular weight of COL1 which interacts with CD133 in IP assay conformed to monomer COL1. Collagens with different structures have inconsistent biological functions.^[^ ^48^ ^]^

PI3K/Akt pathway maintains the self‐renewal and tumorigenesis capacities of cancer stem cells.^[^ ^49^ ^]^ Nevertheless, the mechanism by which the Akt pathway maintains the properties of CSCs remains incompletely understood. Our study confirmed that Akt up‐regulated SLC1A5, which promoted the self‐renewal of glioma stem cells. This finding sheds new light on the mechanism by which the PI3K‐Akt signaling axis enhances the glioma stem cell properties. Akt pathway also regulates Oct4 and Sox2 expression, which are essential transcription factors for maintaining the self‐renewal of cancer stem cells.^[^ ^50^ ^]^ We speculate that the upregulation of SLC1A5 is only one of the pathways by which the COL1‐CD133 interaction enhances the properties of glioma stem cells. In addition, several studies have proved that Akt pathway up‐regulates ATF4 expression through mTOR or NRF2.^[^ ^51^ ^]^ The mechanisms of up‐regulation of ATF4 by Akt pathway in GSCs needs further examination.

Another interesting finding is that the knock down of SLC1A5 reduces Gln uptake in GSCs without obviously changing the level of intermediate metabolites of the TCA cycle, α‐ketoglutarate (α‐KG) and acetyl‐CoA. Consistent with this, deletion of SLC1A5 decreases leukemia initiation and maintenance without reducing the intermediate metabolites of the TCA cycle.^[^ ^31^ ^]^ The mitochondrial oxidation of pyruvate derived from glucose is a major source of acetyl CoA for the TCA cycle. One explanation for this phenomenon is that glioma stem cells take up more glucose than non‐glioma stem cells.^[^ ^52^ ^]^

## 4 Conclusion

In conclusion, the present study identifies COL1 as an extracellular binding molecule of CD133 and defines a pathway of the extracellular matrix microenvironment regulating the characteristics of GSCs. Given the fact that the COL1‐CD133 interaction promotes the tumorigenesis of GSCs, further investigation of this pathway, including isolation of the inhibition of CD133‐COL1 interaction fragment, should provide an approach to anti‐GBM therapy.

## 5 Experimental Section

### Materials and Methods

Anti‐GAPDH Ab was purchased from Santa Cruz Biotechnology. Anti‐Akt Ab, anti‐p‐Akt (Thr308) Ab, anti‐PI3‐kinase p85 Ab, anti‐mouse‐HRP secondary Ab, anti‐His antibody anti‐SLC1A5 antibody, anti‐goat‐HRP secondary Ab, anti‐ATF4 antibody, and anti‐rabbit‐HRP secondary Ab were purchased from Cell Signaling Technology. Anti‐COL1A1 antibody and anti‐alpha‐SMA antibody were purchased from Abcam. Biotinylated Concanavalin A (Con A) and Biotinylated PHA‐L (Phaseolus Vulgaris Leucoagglutinin) were purchased from Vector Laboratories.

### Isolation of GSCs from GBM Tissues

GSCs were isolated from surgical human GBM tissues in accordance with protocols approved by the Fudan University Institutional Review Broads. Briefly, tumor specimens were washed and enzymatically dissociated into single cells. The isolated tumor cells were briefly placed in serum‐free Dulbecco's modified Eagle's and F12 media (DMEM/F12) supplemented with B27 lacking Vitamin A (Invitrogen). GSCs and DGCs were separated through magnetic cell sorting with CD133 Cell Isolation Kit (Miltenyi Biotec). Magnetic separation was performed at least twice. To ectopic expression of SLC1A5‐FLAG in GSCs, GSCs were infected with lentivirus in the presence of 8 µg ml^−1^ polybrene (Sigma‐Aldrich).

### Human and Mouse Tissues

Following informed consent, tumor samples classified as GBM based on World Health Organization (WHO) criteria were obtained from patients undergoing surgical treatment at Zhongshan Hospital in accordance with the appropriate Institutional Review Broads. All mice experiments were performed according to Fudan University guidelines of the Anima Use and Care Committees.

### Glutamine Uptake Assay

The glutamine uptake assay in GSCs was performed as previously described.^[^ ^53^ ^]^ Briefly, cells were grown in Gln‐free DMEM for at least 4 h. Then, 2 mM (U‐^13^C5)‐Gln (Cambridge Isotope Labs) were then added to the medium. Cells were harvested in 80% methanol, and lysates were stored at −80 °C until further analysis by gas chromatography‐mass spectrometry (GC‐MS) for mass isotopomer distribution of various metabolites.

### Metabolomics Analysis of Amino Acids

Cells were collected by scraping in 500–800 µL of −80 °C precooled 1:1 methanol: acetonitrile. Metabolites were extracted by freeze‐thawing three times in liquid nitrogen. After overnight incubation at −80 °C, samples were centrifuged at 13 000 g for 20 min at 4 °C. The supernatants were dried in a vacuum centrifugal concentrator for 3 h (SCIENTZ‐10LS, Scientz, China). The dry extracts were re‐suspended in ddH_2_O and the samples were analyzed with a ACQUITY UPLC H‐Class System (Waters Corporation, USA) coupled to an API 6500 Qtrap Mass Spectrograph (Sciex, USA).

### Plasmids

To knock down endogenous CD133 expression, the CD133 shRNA lentivirus vectors were generated as previously described.^[^ ^8b^ ^]^ The prokaryotic expression plasmid GST‐CD133(20‐108) or its deletion mutant was constructed by inserting the CD133 cDNA sequence (20‐108 amino acids) or its fragment into the BamHI/XhoI sites of the pGEX‐6p‐1 vector. For ectopic expression of CA‐Akt, the LV‐CA‐Akt plasmid was constructed by inserting myr‐Akt cDNA into the LV‐FLAG lentivirus vector. The eukaryotic expression plasmid pcDNA3.1‐IL2 signal peptide‐COL1A1(972‐1206) was constructed by inserting the COL1A1 cDNA sequence (972‐1206 amino acids) or its fragment into the pcDNA3.1‐IL2 signal peptide vector. To construct pGL3‐SLC1A5 plasmid, SLC1A5 core promoter region between −700 and +25 was prepared by PCR amplification of human genomic DNA. Following digestion with restriction enzymes, the fragment was cloned into the pGL3‐Basic vector (Promega), and the correct insertion was confirmed by sequencing.

### Protein Expression and Purification

To expression His‐COL1A1(972‐1206) or its deletion mutant, the expression vector pcDNA3.1‐IL2 signal peptide‐COL1A1(972‐1206) or its deletion mutant was transfected into the HEK293F cells, using polyethyleneimine (PEI)‐mediated transfection. HEK293F cells were cultured in Freestyle 293F expression medium (Sino Biological, Beijing, China) for 7 days. His‐COL1A1(972‐1206) or its mutant were purified from the supernatants using Nickel Beads (Smart Life Sciences).

pGEX‐6P‐1 or pGEX‐6p‐1‐CD133(20‐108) plasmids were transfected into BL21(DE3). BL21 were grown in LB with shaking at 200 rpm, 37 °C until the OD600 reached 0.6. IPTG (1 mm) was added for 5 h at 30 °C to induce protein expression. The GST fusion protein was easily purified by affinity chromatography using Glutathione‐Superflow Resin (Smart Life Sciences).

### In Vivo Tumor Formation Assays

Intracranial transplantation of GSCs into male SCID mice was performed in accordance with a Fudan University Institutional Animal Care and Use Committee approved protocol concurrent with national regulatory standards. Briefly, 72 h after lentivirus infection, cells were counted and certain number cells at the indicated cell density were intracranially injected into mice. Mice were maintained up to 60 days, 90 days, or 180 days or until the development of neurologic signs that significantly inhibited their quality‐of‐life (e.g., ataxia, lethargy, seizures, inability to feed, etc.). After sacrifice, the brains of mice were collected, fixed in 4% paraformaldehyde (PFA), paraffin embedded and sectioned. Tumor formation was determined by systematic histological analysis of H&E‐stained sections.

### Chromatin Immunoprecipitation (ChIP)

ChIP assay was performed to analyze the binding of ATF4 to SLC1A5 promoter region in GSCs using Chromatin immunoprecipitation (ChIP) assay kit (Upstate). Briefly, GSCs from patient's tissues were cross‐linked by addition of 1% formaldehyde for 10–15 min at 37 °C. Cells were washed with cold PBS for three times. Cells were then re‐suspended in an SDS lysis buffer (1% SDS, 50 mm Tris at pH 8, 20 mM EDTA) and were sonicated for five times to shear DNA. Supernatant was collected and diluted in dilution buffer and were carried out overnight with 2 µg of ATF4 antibody or 2 µg of normal mouse IgG at 4 °C. After immunoprecipitation, protein G beads were added to each sample for 4 h, and the beads were then washed. DNA was eluted twice with 100 µL of TE with 1% SDS for 10 min at 65 °C. The cross‐links were reversed overnight at 65 °C. DNA was recovered by phenol extraction and ethanol precipitation. Immunoprecipitated DNA was analyzed for the presence of the ATF4 binding to the SLC1A5 promoter by Real‐time PCR.

### Co‐immunoprecipitation

Co‐immunoprecipitation experiments were performed as previously described.^[^ ^8b^ ^]^ Briefly, GSCs (5 × 10^6^) were collected and lysated in a modified RIPA buffer (1% Triton X‐100, 50 mM Tris (pH = 7.4), 150 mm NaCl, 2 mm EDTA, protease inhibitor cocktail, 25 mm β‐glycerophosphate, 0.2 mm Na_3_VO_4_, 1 mm NaF). Proteins were co‐immunoprecipitated with anti‐CD133 antibody (W6B3C1). Western analysis of electrophoresed proteins was performed using indicated antibodies.

### GM1 – Ganglioside Receptor Binding Assay

Ganglioside Receptor Binding Assay was performed as previously described.^[^ ^54^ ^]^ Briefly,

wells of the 96‐well plate (Corning) were coated overnight at 4 °C with GM1 ganglioside (Sigma). After Washed thrice with 1 × PBST, the wells were then blocked with 200 ml of PTM for 2 h at room temperature. Then, wells were washed thrice with 1 × PBST. GST or GST‐(20‐108) and/or COL1 diluted in ELISA coating buffer were coated on to the plate and incubated for 2 h at 37 °C. The plate was washed again as stated above and incubated with anti‐GST antibody (1:3000 dilution) for 1 h at 37 °C. Next, the plate was incubated with secondary HRP‐conjugated goat anti‐rabbit IgG (Santa Cruz Biotechnology). Following washing with 1× PBST, TMB substrate was added and incubated for 10 min at room temperature. The reaction was terminated by adding 50 ml of 2 N H2SO4 per well and the absorbance was read on a plate reader at 450 nm using a microplate reader.

### GST Pull‐Down Analysis

In GST pull‐down experiment, 10 µg purified GST or GST‐CD133(20‐108) protein with GST‐beads from bacteria BL21 were incubated with His‐COL1A1(972‐1206) protein for 1 h at 4°C. After washing with RIPA (1% Triton X‐100, 50 mm Tris (pH = 7.4), 150 mm NaCl, 2 mm EDTA, protease inhibitor cocktail, 25 mm β‐glycerophosphate, 0.2 mm Na_3_VO_4_, 1 mm NaF) for three times, the pull‐down products were analyzed by Coomassie Blue staining and western blotting using anti‐His antibody.

### Cell Cultures

The sorted GSCs were cultured in the DMEM/F12 media supplemented with B27 lacking vitamin A (Invitrogen), 2 µg ml^−1^ heparin (Sigma), 20 ng ml^−1^ EGF (Chemicon), and 20 ng ml^−1^ FGF‐2 (Chemicon) for a short period before treatment and analysis. DGCs were plated in DMEM with 10% fetal bovine serum for at least 12 h to permit cell survival. Prior to performing experiments with DGCs, DMEM with 10% fetal bovine serum was replaced with supplemented DMEM/F12 media in order for experiments to be performed in identical media. The isolation and culture of CAFs were performed as previously described.^[^ ^55^ ^]^ 293T cells were cultured in DMEM media supplemented with 10% FBS (fetal bovine serum) (Gbico).

### Immunofluorescence

For immunostaining analysis of the contact of GSCs to COL1, GBM sections were fixed with 4% PFA for 20 minutes at room temperature, washed three times with PBS, and then blocked with a PBS‐based solution containing 5% normal goat serum and 0.3% Triton X‐100. Cells were co‐incubated overnight at 4 °C with goat polyclonal anti‐COL1A1 antibody (Abcam) or SLC1A5 (CST) and mouse monoclonal anti‐CD133 (W6B3C1 clone) (Miltenyi Biotec). After washed three times with PBS, cells were co‐incubated with Alexa 488 conjugated donkey anti‐mouse IgG (Invitrogen; 1:400) and Alexa 594 conjugated donkey anti‐rabbit IgG (Invitrogen, 1:800). Nucleus were counterstained with DAPI (Sigma; 10 µg ml^−1^). Immunofluorescent images were collected on a Leica TCS SP5 confocal microscope and analyzed using LAS AF software.

### Immunohistochemistry

Paraffin‐embedded sections were processed for immunohistochemical staining as previously documented.^[^ ^8b^ ^]^ Sections were incubated with primary antibodies: type I collagen, SLC1A5 followed by HRP‐conjugated secondary antibody (Santa Cruz Biotechnology). The immunoreactivity was visualized with 3, 3‐diaminobenzidine (Dako). Sections were counterstained with hematoxylin (Sigma). The scores for semiquantitative staining of the tissue sections were was obtained by combining the score of the percentage of positive cells and the score of the average staining intensity in ten randomly selected fields per section.^[^ ^56^ ^]^ Picrosirius red staining was performed as previously described.^[^ ^33^ ^]^

### Dual Luciferase Assay

Cells were co‐transfected with pGL3‐SLC1A5 containing SLC1A5 core promoter region (−700 to +25) or its deletion mutants and control or CA‐Akt plasmids and internal control pRL plasmids. 48–72 h after transfection, cells were rinsed in PBS and lysed in a Passive Lysis Buffer (Promega). The luciferase activities were measured using Dual‐Luciferase Reporter Assay System (Promega) with Turner luminometer and normalized to the Renilla Luciferase activity for transfection efficiency. Data were represented as the mean from three independent experiments.

### Dataset and Bioinformatics Analyses

Rembrandt, Gravendeel and TCGA datasets and corresponding clinical information were obtained from GlioVis (http://gliovis.bioinfo.cnio.es/). Count reads from the raw RNA‐sequencing datasets were normalized and transformed in log2 scale for statistical analysis. Different expression of COL1A1 across glioma I–IV grades were identified using GlioVis online tool. In TCGA GBM dataset, pre‐defined GSC markers and COL1A1 expression values were extracted. Pearson's correlation coefficient or Spearma's rank correlation coefficient was calculated to find expression correlation between COL1A1 and markers of fibroblasts. The statistical computations and visualization were performed using R version 4.0.3 (R Foundation for Statistical Computing, Vienna, Austria).

### COL1 Solution Treatment

COL1 prepared with hydrochloric acid was purchased from Sigma.  mL^−1^ COL1 solution was directly added to the cell culture system. To examine the effect of COL1 on the sphere formation of GSCs, GSCs were treated COL1 solution for 10 days. To examine the effect of COL1 on the activity of PI3K or Akt phosphorylation level, GSCs were treated COL1 solution for 6 h.

### Neurosphere Formation Assay

For single cell neurosphere formation assay, cells were trypsinized and single‐cell suspensions were cultured in 24‐well plates (100 cell per well) containing supplemented DMEM/F12 medium and 2 ng ml^−1^ EGF and 2 ng ml^−1^ FGF. After 10 days, the number of neurospheres per well were quantified.

For neurosphere formation assay of cells treated with COL1, either vehicle control or COL1 solution was added every three days. After 10 days, the number of neurospheres per well were quantified.

### Preparation of Patient‐Derived ECM Hydrogel Formation

Preparation of patient‐derived ECM were prepared as previously described.^[^ ^57^ ^]^ Patient tissues were cut into small pieces. Subsequently, decellularizing solution (0.1% (v/v) ammonium hydroxide and 1% (v/v) Triton X‐100) was treated for 2 days to remove the cellular component from patient‐derived brain tissues. Next, decellularized brain tissue‐derived brain ECM (pdECM) was washed using distilled water to remove the detergent solution and cellular residue. Finally, pdECM was lyophilized and stored at −20 °C until use.

### Western Blot Analysis

Western blot analysis was performed as previously described.^[^ ^9^ ^]^ Primary antibodies included: mouse monoclonal anti‐CD133 (W6B3C1 clone) (Miltenyi Biotec), rabbit monoclonal anti‐GAPDH Ab (Cell signaling), anti‐Akt Ab (Cell signaling), anti‐p‐Akt (Thr308) Ab (Cell signaling), anti‐PI3‐kinase p85 Ab (Millipore), and anti‐SLC1A5 antibody (Cell signaling.

### Statistical Analysis

In general, significance was tested by unpaired two‐tailed Student's *t*‐test using GraphPad InStat 5.0 software. *P*‐values < 0.05 were considered statistically significant. Results are expressed as the mean ± standard deviation (SD) or mean ± standard error of the mean (mean ± SEM). The Kaplan–Meier method was used to determine survival probability and differences were assessed by the log‐rank test.

## Conflict of Interest

The authors declare no conflict of interest.

## Author Contributions

Y.W., S.G., Y.S., and Y.Y. contributed equally to this work. Y.W. designed the study and methodology; performed data collection, analysis, and validation; wrote, and edited the manuscript; and wrote the original draft. S.G., Y.S., and Y.Y. designed methodology; performed collection, analysis, and interpretation of data. Q.C., S.H., X.C., and W.X. performed data collection, analysis, and interpretation of data. Y.L. and J.J. performed supervision; funding acquisition, and conceptualization; designed methodology; performed validation; wrote, reviewed, and edited the final manuscript; and wrote the original draft

## Supporting information

Supporting Information

## Data Availability

The data that support the findings of this study are available from the corresponding author upon reasonable request.
