# Supplementary material for: The Interaction between Collagen 1 and High Mannose Type CD133 Up‐Regulates Glutamine Transporter SLC1A5 to Promote the Tumorigenesis of Glioblastoma Stem Cells
Source: Adv Sci (Weinh). 2023 Nov 23;11(3):2306715. doi: 10.1002/advs.202306715 (PMC10797482; doi:10.1002/advs.202306715)
Supplement: Supplementary file 1 — Supporting Information [file ADVS-11-2306715-s001.pdf]

ADVANCED SCIENCE
Open Access

Supporting Information

for *Adv. Sci.*, DOI 10.1002/advs.202306715

The Interaction between Collagen 1 and High Mannose Type CD133 Up-Regulates Glutamine Transporter SLC1A5 to Promote the Tumorigenesis of Glioblastoma Stem Cells

Yuanyan Wei, Shuting Geng, Yu Si, Yuerong Yang, Qihang Chen, Sijing Huang, Xiaoning Chen, Wenlong Xu, Yinchao Liu* and Jianhai Jiang**

The interaction between Collagen 1 and high mannose type CD133 up-regulates glutamine transporter SLC1A5 to promote the tumorigenesis of glioblastoma stem cells

Yuanyan Wei[1#], Shuting Geng[1#], Yu Si[1#], Yuerong Yang[1#], Qihang Chen[1], Shijing Huang[1], Xiaoning Chen[1], Wenlong Xu[2], Yinchao Liu[3]*, Jianhai Jiang[1]*

[1]NHC Key Laboratory of Glycoconjuates Research, Department of Biochemistry and Molecular Biology, School of Basic Medical Sciences, Fudan University, Shanghai 200032, People's Republic of China

[2]Department of Surgery, Zhongshan Hospital, Fudan University, Shanghai 200032, People's Republic of China

[3]Department of Neurosurgery, Shandong Provincial Hospital Affiliated to Shandong First Medical University, Jinan, Shandong 250021, People's Republic of China

This file includes:

Supplementary Figure S1-S7 with Legends

Supplementary Table S1-S2

Supplemental Information Inventory

Figure S1. Extracellular COL1 binds to CD133 in GSCs, related to Figure 1.

Figure S2. COL1 is a potential niche component for CD133-positive glioblastoma cells, related to Figure 2.

Figure S3. The interaction between COL1 and CD133 promotes the self-renewal of GSCs, related to Figure 3.

Figure S4. The interaction between COL1 and CD133 activates PI3K-Akt pathway, related to Figure 4.

Figure S5. The interaction between COL1 and CD133 up-regulates SLC1A5 in GSCs, related to Figure 5.

Figure S6. Down-regulation of SLC1A5 inhibited the effect of COL1 on the tumorigenesis of GSCs, related to Figure 6.

Figure S7. The level of COL1 was positively correlated with glioma grades, related to Figure 7.

Table S1. Screening protein interacting with CD133 N-terminal (aa20-108) using the yeast two hybrid system, related to Figure 1

Table S2. Pathologic characteristics of Patient #1-#3 brain tumors, related to Figure 1

Figure S1

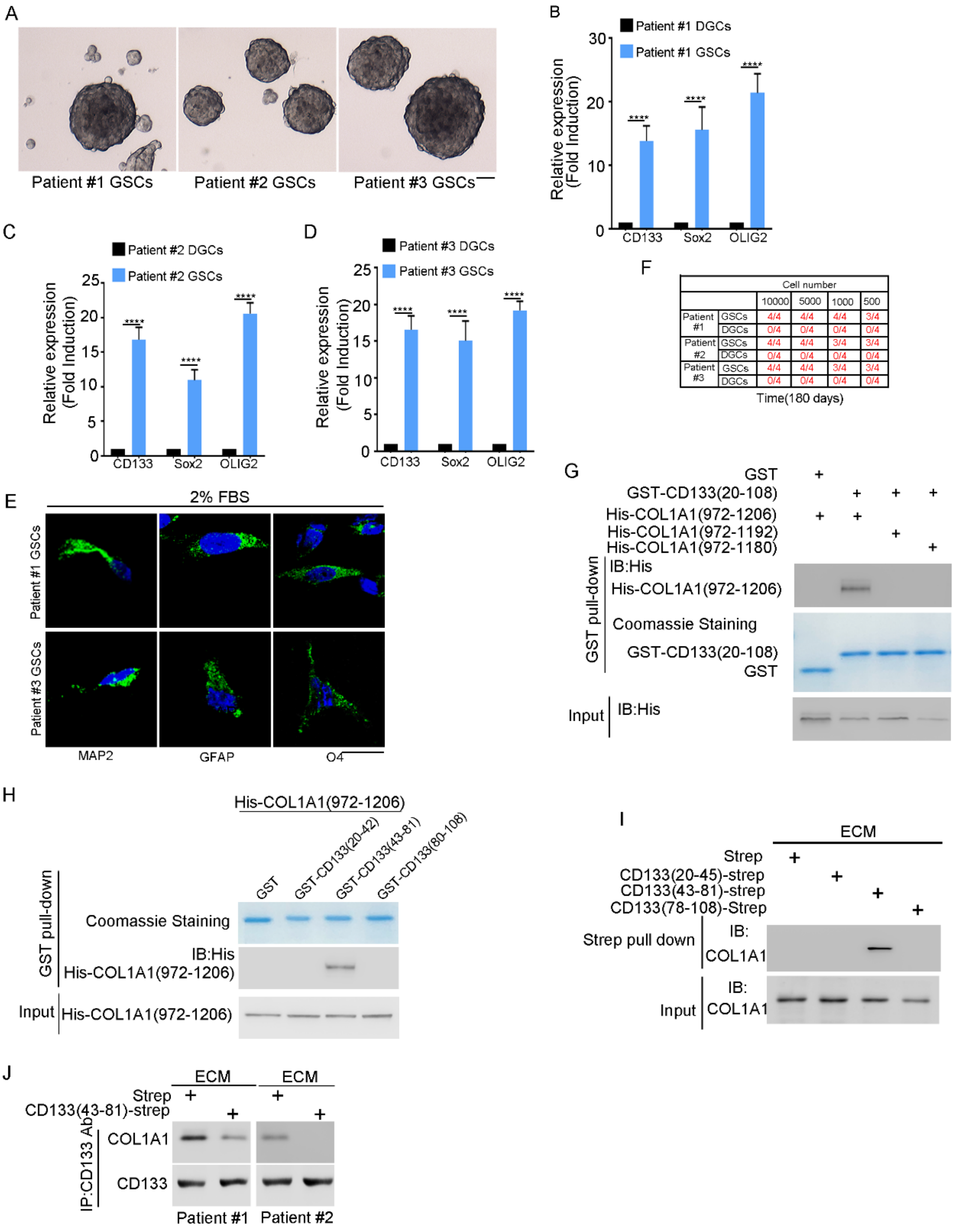

Figure S1. Extracellular COL1 binds to CD133 in GSCs, related to Figure 1.

A. Representative images of neurospheres derived from GSCs isolated from glioblastoma specimens (patients #1-#3). Scale bar, 50 μM.

B-D. QRT-PCR analysis of stem cell marker CD133, Sox2 and OLIG2 mRNA level in the matched GSCs and DGSc cells isolated from patient #1 (B), patient #2 (C) and

patient #3 (D) GBM tissues.

E. Multilineage differentiation capacity of GSCs was evaluated by staining for the O4 (oligodendrocytic marker), the MAP2 (neuron marker) and the GFAP (astrocyte marker). Nucleus were counterstained with DAPI. “2% FBS” indicates the addition of serum. Scale bars represent 10 µM.

F. An in vivo limiting dilution tumor formation assay (employing 10,000, 5,000, 1,000 or 500 cells per mouse) was performed to compare the tumor-initiating capacity of GSCs with matched DGCs. Mice were sacrificed when they were moribund or 180 days after implantation. Tumor formation was determined by histology. The table displays the number of mice developing tumors.

G. GST or GST-CD133(20-108) proteins were incubated with purified His-COL1A1 (972-1206) protein and its deletion mutant. The GST pull-down products were blotted with anti-His antibody. GST and GST-CD133(20-108) were shown by Coomassie Blue staining.

H. GST, GST-CD133(20-42), GST-CD133(43-81), GST-CD133(80-106) proteins were incubated with purified His-COL1A1 (972-1206) protein. The GST pull-down products were blotted with anti-His antibody. GST, GST-CD133(20-42), GST-CD133(43-81), GST-CD133(80-106) were shown by Coomassie Blue staining.

I. Strep peptide or strep-tagged CD133 N-terminal deletion mutant were incubated with ECM. The Strep pull-down products were blotted with anti-COL1A1 antibody.

J. The effect of Strep or CD133(43-81)-Strep on the interaction between CD133 and COL1 in vivo. The lysates of GSCs pretreated with ECM and/or Strep or CD133(43-81)-Strep were subjected to IP using anti-CD133 (Clone W6B3C1) antibody, followed by immunoblotting with anti-CD133 antibody or anti-COL1A1 antibody.

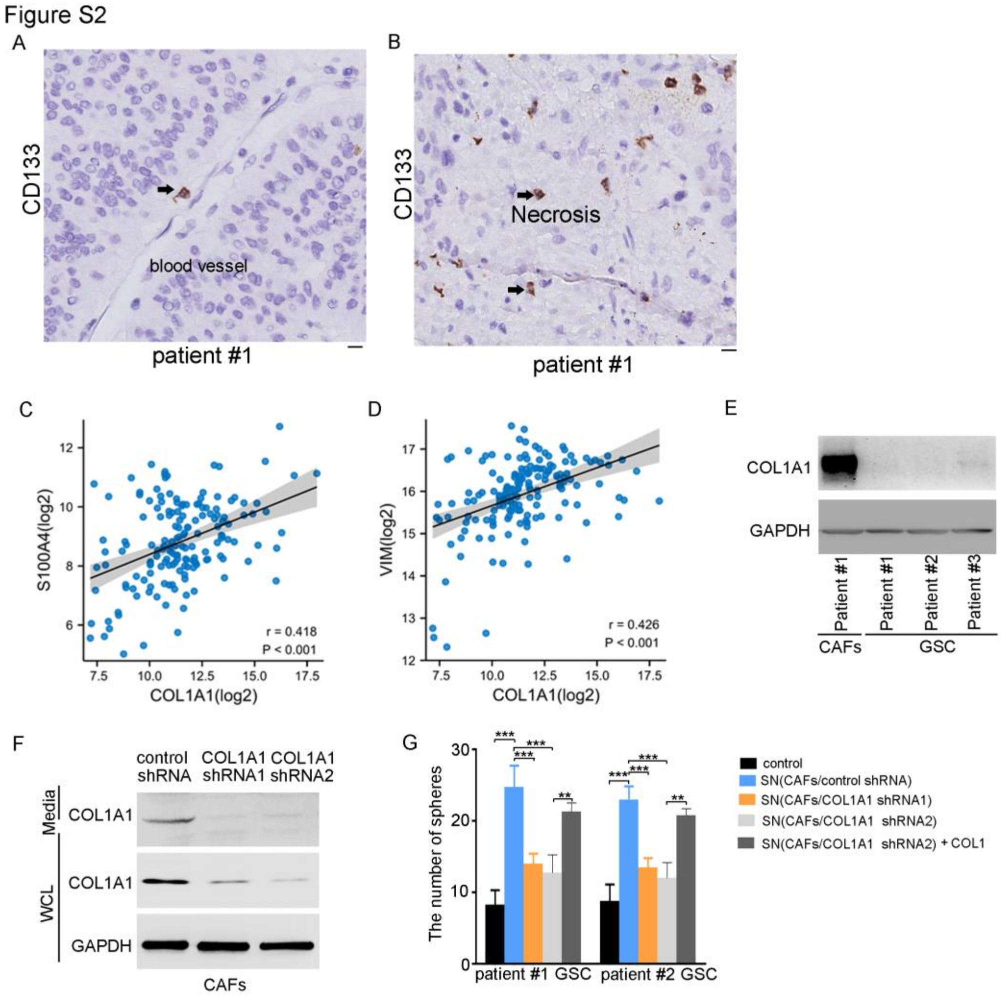

Figure S2. COL1 is a potential niche component for CD133-positive glioblastoma cells, related to Figure 2.

A-B. Representative immunohistochemical staining of CD133 in primary GBM tissue. GSCs were located in vascular niche (**A**) and necrosis area (**B**). Scale bars, 10 μM.

C-D. The correlation between COL1A1 expression with fibroblast marker markers S100A4 (**C**) and VIM (**D**) in TCGA database was indicated. Numbers represent coefficient values (n = 160).

E. Western blot analysis of COL1A1 expression in fibroblasts and patient #1-#3 GSCs. GAPDH expression served as a loading control.

F. Western blot analysis of COL1 expression in CAFs expressing control or COL1 shRNA. GAPDH expression served as a loading control.

G. Single cell sphere formation assay of GSCs treated with supernatant from CAFs expressing control shRNA or COL1A1 shRNA and/or COL1. Results are expressed as mean ± SD (n = 4; ****p* < 0.001, ***p* < 0.01).

Figure S3

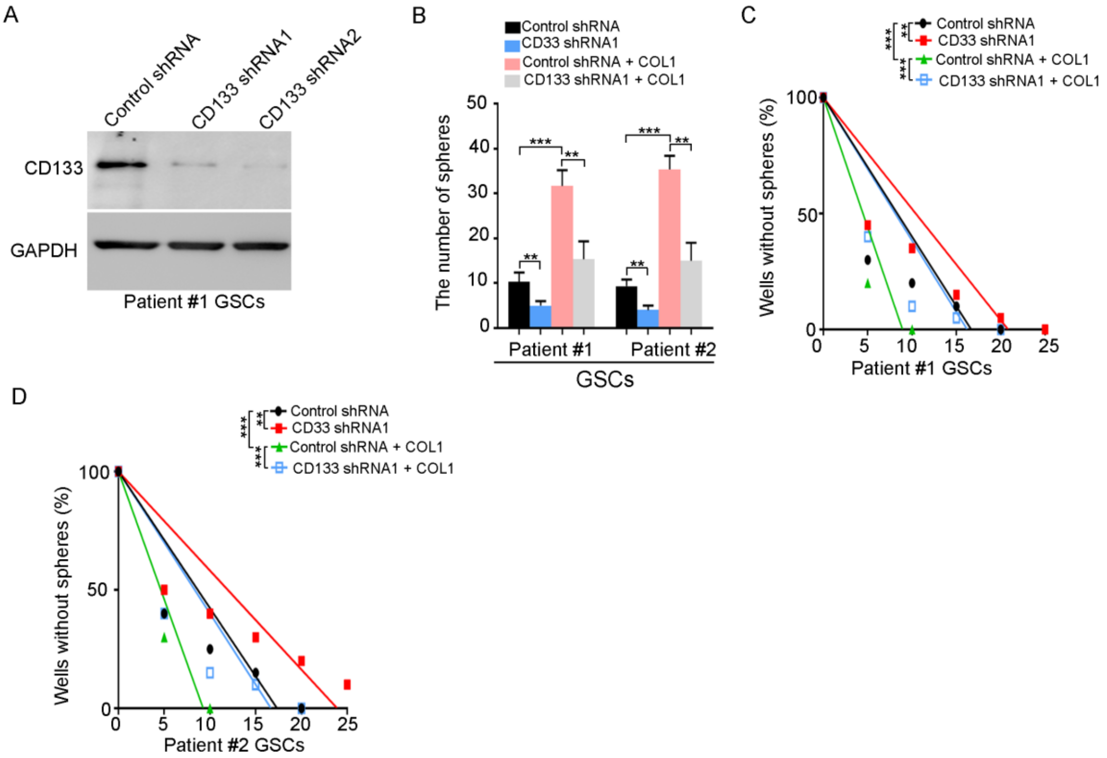

Figure S3. The interaction between COL1 and CD133 promotes the self-renewal of GSCs, related to Figure 3.

A. Relative level of CD133 expression in GSCs expressing control shRNA or CD133 shRNA1-2.

B. Single cell sphere formation assay of GSCs expressing control shRNA or CD133 shRNA1 treated with COL 1. Results are expressed as mean ± SD (n = 4; ***p* < 0.01, ****p* < 0.001).

C-D. Limiting dilution assay shows that the COL1-CD133 interaction increased stem cell frequency in Patient #1 (C) and Patient #2 (D) GSCs. n=10, ***p* < 0.01, ****p* < 0.001 by ELDA analysis.

Figure S4

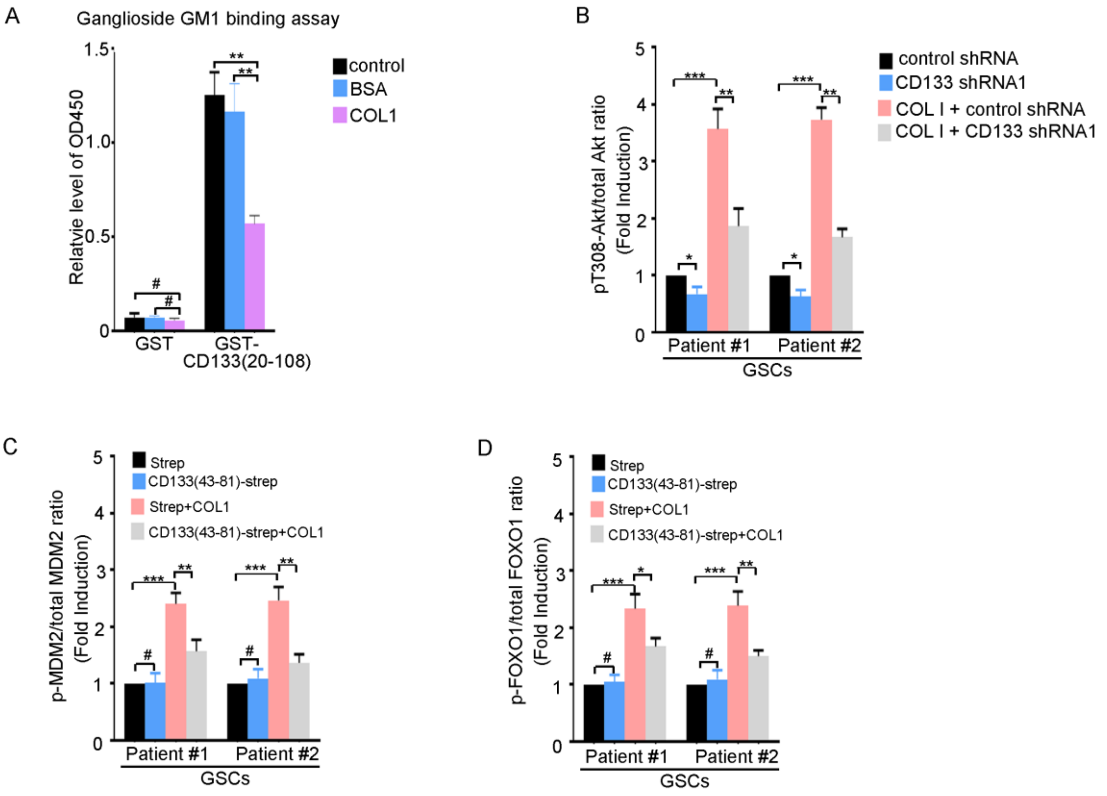

Figure S4. The interaction between COL1 and CD133 activates PI3K-Akt pathway, related to Figure 4.

A. Ganglioside GM1 ELISA binding assay analysis of the effect of COL1 on the binding of GST or GST(20-108) to GM1 in the presence of COL1 or control. Results are expressed as mean ± SD (n = 3; **p* < 0.05, ***p* < 0.01, #, n.s).

B. GSCs expressing control shRNA or CD133 shRNA1 were treated with COL 1 (10 μg/ml). Whole-cell lysates were analyzed by western blot. The relative densities of pT308-Akt to total Akt were quantified using densitometry. Values are normalized to that of GSCs treated with control. Results are expressed as mean ± SD from three separate experiments; **p* < 0.05, ***p* < 0.01, ****p* < 0.001.

C-D. The level of MDM2 (C) and FOXO1 (D) phosphorylation in GSCs pre-treated with COL 1 (10 μg/ml) and Strep or CD133(43-81)-Strep. Whole-cell lysates were analyzed by western blot. GAPDH was blotted as a loading control. Values are normalized to that of GSCs treated with control. Results are expressed as mean ± SD from three separate experiments; **p* < 0.05, ***p* < 0.01, ****p* < 0.001.

Figure S5

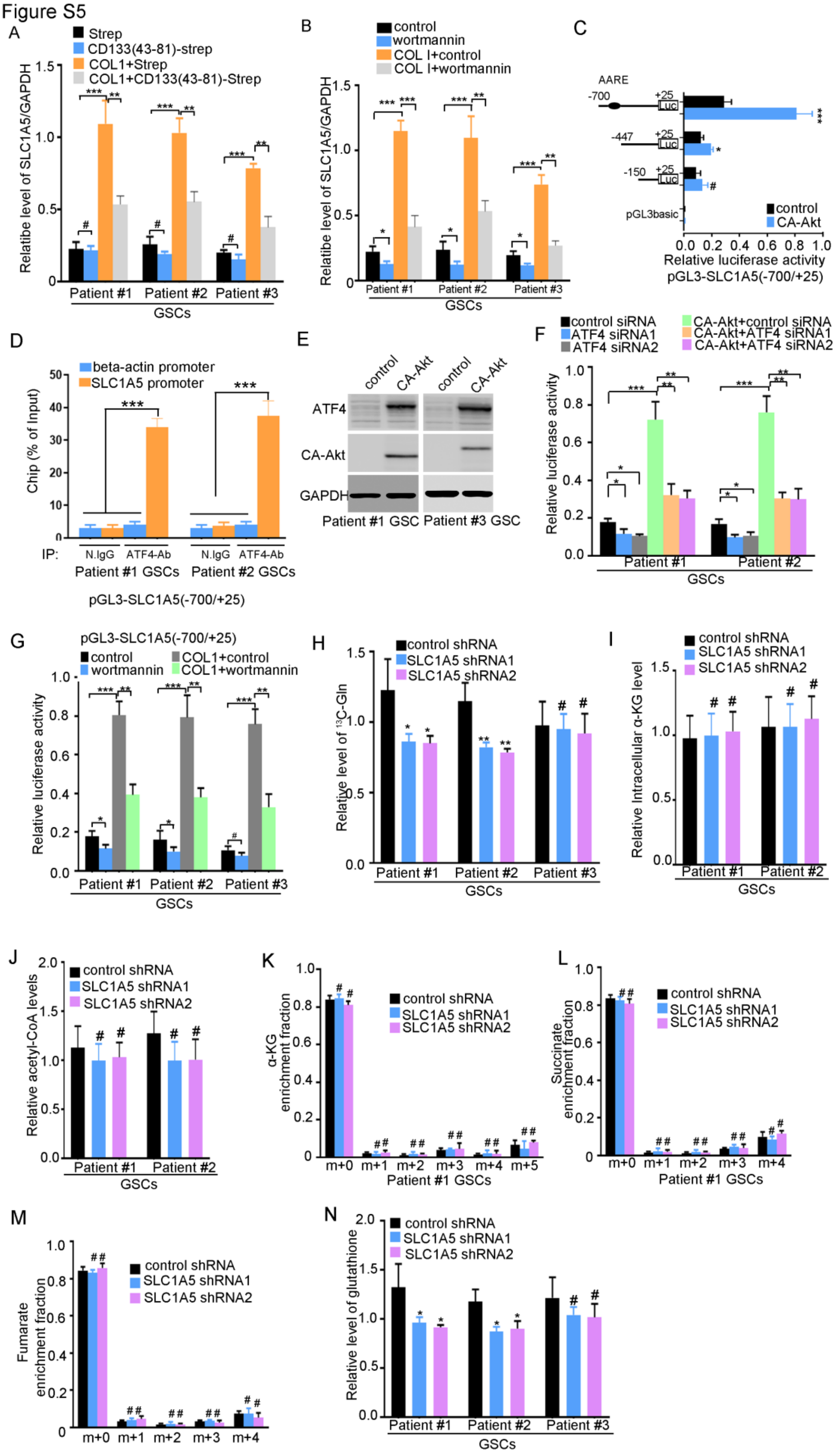
A
Strep
CD133(43-81)-strep
COL1+Strep
COL1+CD133(43-81)-Strep
Relatibe level of SLC1A5/GAPDH
Patient #1 Patient #2 Patient #3
GSCs
B
control
wortmannin
COL I+control
COL I+wortmannin
Relatibe level of SLC1A5/GAPDH
Patient #1 Patient #2 Patient #3
GSCs
C
AARE
control
CA-Akt
pGL3basic
Relative luciferase activity
pGL3-SLC1A5(-700/+25)
D
beta-actin promoter
SLC1A5 promoter
Chip (% of Input)
IP: N.IgG ATF4-Ab N.IgG ATF4-Ab
Patient #1 GSCs Patient #2 GSCs
pGL3-SLC1A5(-700/+25)
E
control CA-Akt control CA-Akt
ATF4
CA-Akt
GAPDH
Patient #1 GSC Patient #3 GSC
F
control siRNA
ATF4 siRNA1
ATF4 siRNA2
CA-Akt+control siRNA
CA-Akt+ATF4 siRNA1
CA-Akt+ATF4 siRNA2
Relative luciferase activity
Patient #1 Patient #2
GSCs
G
pGL3-SLC1A5(-700/+25)
control
wortmannin
COL1+control
COL1+wortmannin
Relative luciferase activity
Patient #1 Patient #2 Patient #3
GSCs
H
control shRNA
SLC1A5 shRNA1
SLC1A5 shRNA2
Relative level of 13C-Gln
Patient #1 Patient #2 Patient #3
GSCs
I
control shRNA
SLC1A5 shRNA1
SLC1A5 shRNA2
Relative Intracellular α-KG level
Patient #1 Patient #2
GSCs
J
control shRNA
SLC1A5 shRNA1
SLC1A5 shRNA2
Relative acetyl-CoA levels
Patient #1 Patient #2
GSCs
K
control shRNA
SLC1A5 shRNA1
SLC1A5 shRNA2
α-KG enrichment fraction
m+0 m+1 m+2 m+3 m+4 m+5
Patient #1 GSCs
L
control shRNA
SLC1A5 shRNA1
SLC1A5 shRNA2
Succinate enrichment fraction
m+0 m+1 m+2 m+3 m+4
Patient #1 GSCs
M
control shRNA
SLC1A5 shRNA1
SLC1A5 shRNA2
Fumarate enrichment fraction
m+0 m+1 m+2 m+3 m+4
N
control shRNA
SLC1A5 shRNA1
SLC1A5 shRNA2
Relative level of glutathione
Patient #1 Patient #2 Patient #3
GSCs

Figure S5. The interaction between COL1 and CD133 up-regulates SLC1A5 in GSCs, related to Figure 5

A-B. The mRNA level of SLC1A5 in GSCs treated with control or COL1 and Strep or CD133(43-81)-Strep (A) or wortamannin and/or COL1was examined (B) by qRT-PCR. Results are expressed as mean ± SD (n = 3; *p < 0.05, **p < 0.01, ***p < 0.001).

C. GSCs cells were transfected with pGL3-SLC1A5(-700/+25) construct or with the truncated SLC1A5 promoter constructs shown above and with or without CA-Akt expression vector. Luciferase activity was normalized to pRL activity.

D. ChIP assay analysis of the binding of endogenous ATF4 to SLC1A5 promoter in GSCs treated with COL1. Immunoprecipitations were carried out with control IgG (N.IgG) or anti-ATF4 antibody. Coprecipitating DNA was revealed by qRT-PCR with the indicated primers.

E. Western blot analysis of ATF4 expression in GSCs expressing control or CA-Akt. GAPDH expression served as a loading control.

F. GSCs transiently transfected with pGL3-SLC1A5(-700/+25) and pRL-SV40 were transiently transfected with control siRNA or ATF4 siRNA and CA-Akt. 72 h later, the luciferase activity was normalized to Renilla luciferase activity. Results are expressed as mean ± SD (n = 3; **p < 0.01, ***p < 0.001).

G. GSCs cells transfected with pGL3-SLC1A5(-700/+25) construct were treated with control or COL I and control or Wortammnin. Luciferase activity was normalized to pRL activity. Results are expressed as mean ± SD (n = 3; **p < 0.01, ***p < 0.001).

H. GSCs expressing control shRNA or SLC1A5 shRNA1 pretreated with control or COL I were incubated with ^{3}C-L-glutamine to assess L-glutamine uptake. Values are normalized to that of GSCs treated with control. Results are expressed as mean ± SD (n = 3; **p < 0.01, *p < 0.05).

I-J. Intracellular α-KG (I) and acetyl-CoA (J) in GSCs expressing control shRNA or SLC1A5 shRNA1 were determined as described in Methods. Results are expressed as mean ± SD (n = 3; #, ns).

K-M. The incorporation of ^{13}C atoms is denoted as m+ n, where n is the number of

13C atoms. Metabolic abundance of ^{13}C-L-glutamine-derived TCA metabolites from GSC cells expressing control shRNA or SLC1A5 shRNA (K) a-KG, (L) succinate, (M) fumarate (n = 3; #, ns).

N. Intracellular glutathione in GSCs expressing control shRNA or SLC1A5 shRNA1 were determined as described in Methods. Results are expressed as mean ± SD (n = 3; *$p < 0.05$, #, ns).

Figure S6

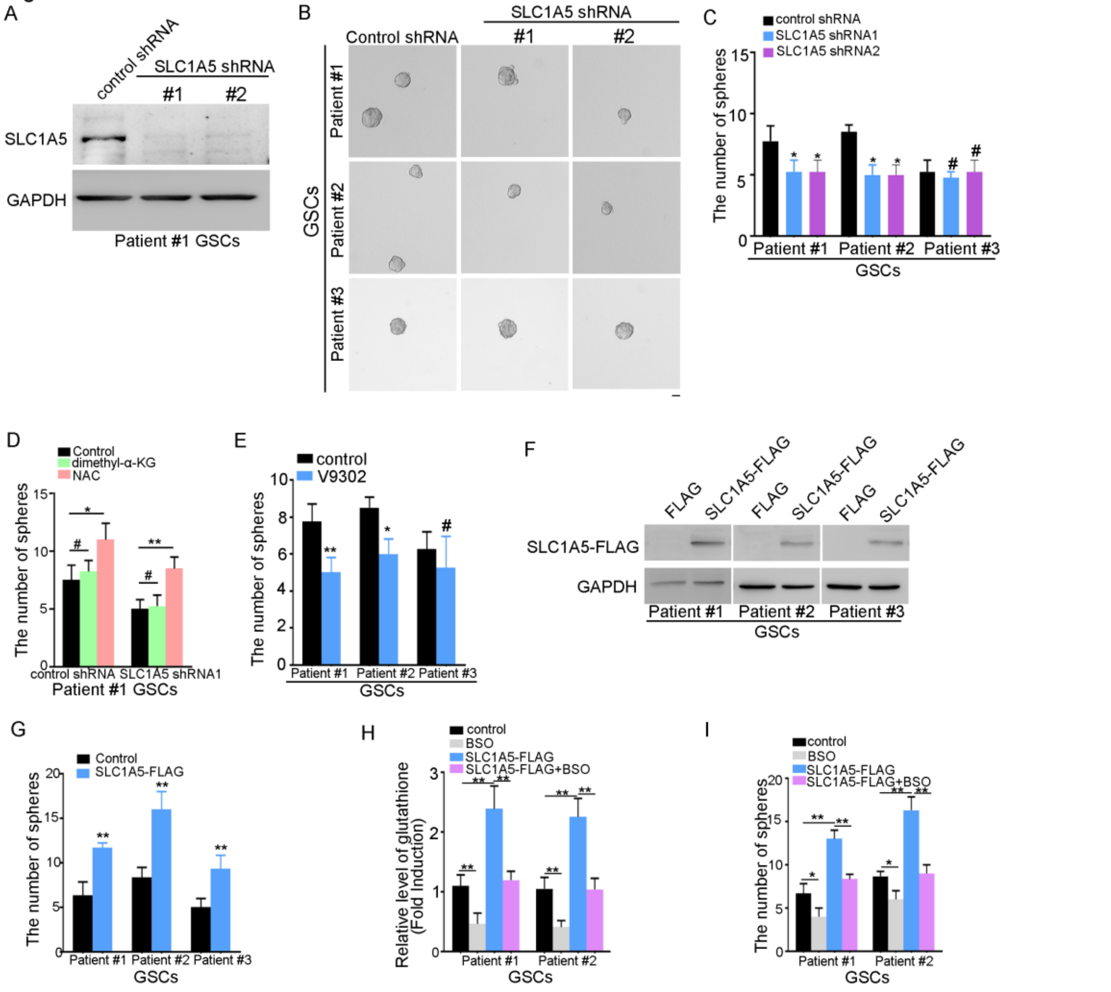

J

		Patient#1 GSCs			Patient#2 GSCs		
	Cell number	5000	1000	500	5000	1000	500
Control shRNA	Incidence	2/4	2/4	1/4	3/4	2/4	1/4
SLC1A5 shRNA1	Incidence	1/4	1/4	0/4	2/4	1/4	0/4
SLC1A5 shRNA2	Incidence	1/4	1/4	0/4	2/4	1/4	0/4

Time (60 days)

L

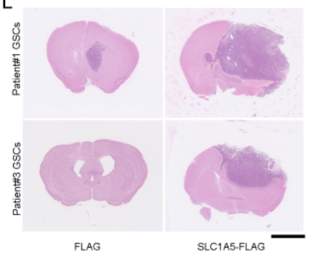

M

		Patient#1 GSCs			Patient#3 GSCs		
	Cell number	5000	1000	500	5000	1000	500
FLAG	Incidence	2/4	2/4	1/4	2/4	2/4	1/4
SLC1A5-FLAG	Incidence	4/4	4/4	2/4	4/4	4/4	3/4

Time (60 days)

K

		Patient#1 GSCs			Patient#2 GSCs		
	Cell number	5000	1000	500	5000	1000	500
control	Incidence	2/4	2/4	1/4	2/4	2/4	1/4
V9302	Incidence	1/4	1/4	0/4	1/4	1/4	0/4

Time (60 days)

N

Patient#1 GSCs		control shRNA			SLC1A5 shRNA1		
	cell number	5000	1000	500	5000	1000	500
Strep	Incidence	2/4	2/4	1/4	1/4	1/4	0/4
CD133(43-81)-Strep	Incidence	2/4	2/4	1/4	1/4	1/4	0/4
COL1+Strep	Incidence	4/4	4/4	3/4	2/4	1/4	1/4
COL1+CD133(43-81)-Strep	Incidence	2/4	2/4	1/4	2/4	1/4	1/4

Time (60 days)

Figure S6. Down-regulation of SLC1A5 inhibited the effect of COL1 on the tumorigenesis of GSCs, related to Figure 6

A. Western blot analysis of SLC1A5 expressing in GSCs expressing control shRNA, SLC1A5 shRNA1-2. GAPDH expression served as a loading control.

B-C. Single cell sphere formation assay of GSCs expressing control shRNA or SLC1A5 shRNA1, SLC1A5 shRNA2. (**B**) Representative images of sphere are shown. Scale bar, 25 μM. (**C**) Results are expressed as mean ± SD (n = 4; *$p < 0.05$, **$p < 0.01$).

D. Single cell sphere formation assay of GSCs treated with control or NAC or dimethyl-α-KG. Results are expressed as mean ± SD (n = 4; *$p < 0.05$, **$p < 0.01$).

E. Single cell sphere formation assay of patient #1-#3 GSCs treated with control or V9302. Results are expressed as mean ± SD (n = 4; *$p < 0.05$, **$p < 0.01$).

F. Western blot analysis of SLC1A5-FLAG expression in GSCs expressing control or SLC1A5-FLAG.

G. Single cell sphere formation assay of GSCs expressing control or SLC1A5 expression vector. Results are expressed as mean ± SD (n = 4; **$p < 0.01$).

H**.** The intracellular glutathione levels in GSCs expressing control or CD133-FLAG treated with control or Buthionine sulphoximine (BSO) were assessed with glutathione colorimetric assay kit. Values are normalized to that of GSCs treated with control. Results are expressed as mean ± SD (n = 3; **$p < 0.01$).

I. Single cell sphere formation assay of patient #1 and patient #2 GSCs expressing control or SLC1A5-FLAG treated with control or Buthionine sulphoximine (BSO). Results are expressed as mean ± SD (n = 3; **$p < 0.01$).

J. An intracranial limiting dilution tumor formation assay (employing 5,000, 1,000, and 500 cells per mouse) was performed using GSCs expressing control shRNA, SLC1A5 shRNA1 and SLC1A5 shRNA2. The table displays the number of mice developing tumors.

K. The tumor-initiating capacity of GSCs treated with control or V9302. An intracranial limiting dilution tumor formation assay (employing 5,000, 1,000, and 500 cells per mouse) was performed. The table displays the number of mice developing tumors.

L-M. The tumor-initiating capacity of GSCs expressing control or SLC1A5-FLAG. An intracranial limiting dilution tumor formation assay (employing 5,000, 1,000, and 500 cells per mouse) was performed. (**L**) H&E staining of mouse brain shows tumors

formation by GSCs. Scale bar, 2.5 mm. (**M**) The table displays the number of mice developing tumors.

N. The tumor-initiating capacity of patient #1 GSCs expressing control shRNA or SLC1A5 shRNA treated with Strep or CD133(43-81)-Strep and control or COL1 (10 μg/ml). An intracranial limiting dilution tumor formation assay (employing 5,000, 1,000, and 500 cells per mouse) was performed using GSCs expressing control shRNA or SLC1A5 shRNA treated with Strep or CD133(43-81)-Strep and control or COL1 (10 μg/ml). The table displays the number of mice developing tumor.

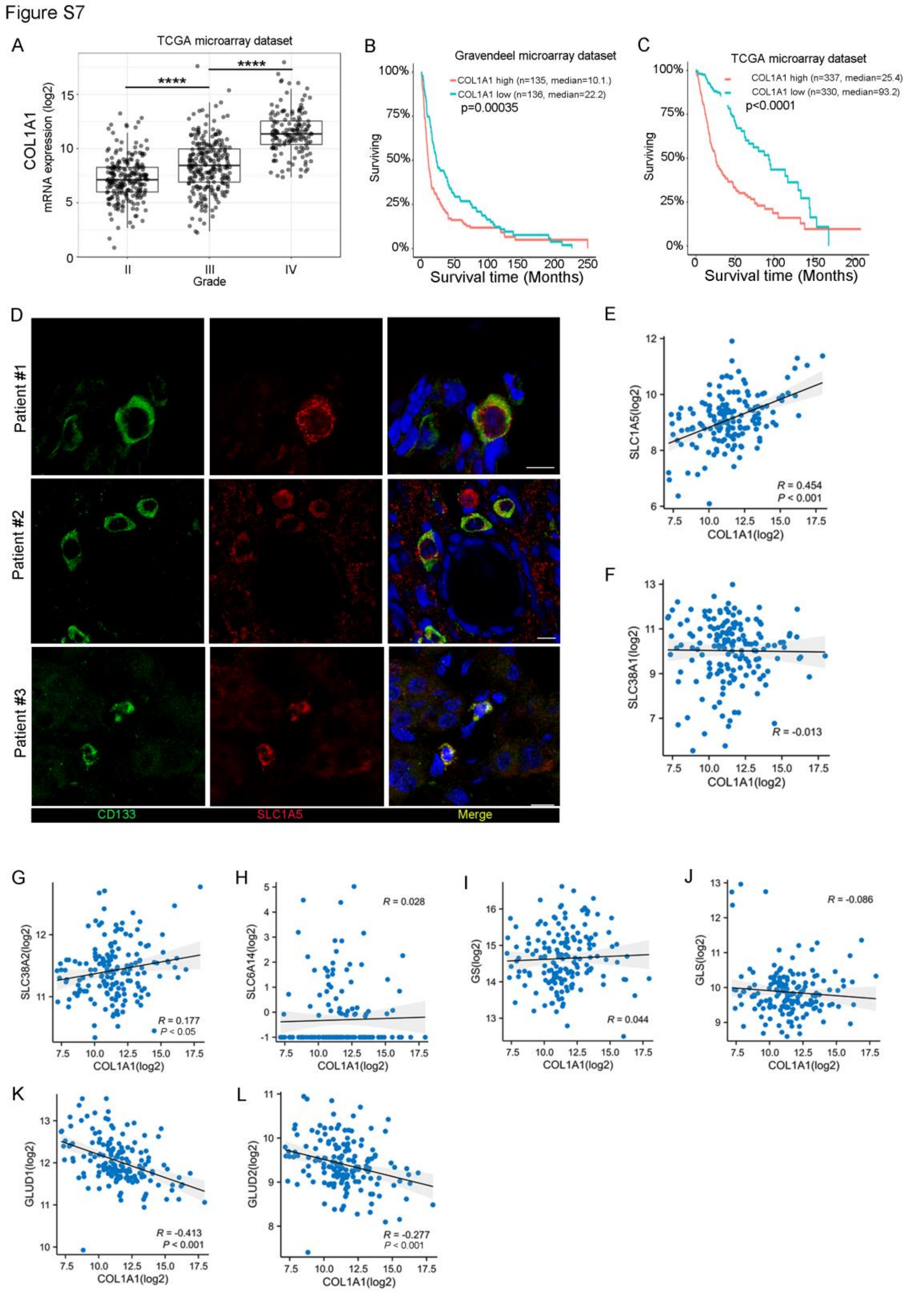

Figure S7. The level of COL1 was positively correlated with glioma grades

related to Figure 7.

A. TCGA analysis of COL1A1 mRNA expression in glioma with different grade.

B-C. Kaplan-Meier analysis of patient cohorts showing survival effects of COL1A1 expression alone. Data was derived from Gravendeel microarray (B) and TCGA microarray (C) using the median mRNA expression of COL1A1 for the corresponding dataset as the cut off.

D. Immunofluorescence of primary human tumor tissue sections revealed co-expression of CD133 and SLC1A5; scale bars represent 10 μM.

E-L. The correlation between COL1A1 expression with glutamine metabolism related genes SLC1A5 (E), SLC38A1 (F), SLC38A2 (G), SLC6A14 (H), GS (I), GLS (J), GLUD1 (K) and GLUD2 (L) in TCGA GBM database was indicated. Numbers represent coefficient values (n = 160). F, H-J, $p > 0 .05$, not significant.

Table S1. Screening protein interacting with CD133 N-terminal extracellular domain (aa20-108) using the yeast two hybrid system, related to Figure 1

N-terminal extracellular domain of CD133 (residues 20-108) was used as the bait for yeast two-hybrid screen. 4 positive clones were obtained from at least 1×10^6 clones of a human fetal brain library.

**Clone**	**Gene Name**	**Domain**	**Gene Function**	**Validation by IP in GSC**
1	COL1A1	972-1206	collagen type I alpha 1 chain	Yes
2	COPS5	80-200	regulator of the ubiquitin	No
3	LEMD3	85-198	LEM domain containing 3	No
4	ID2	53-95	inhibitor of DNA binding 2	No

Table. S2. Pathologic characteristics of Patient #1-#3 brain tumors, related to Figure 1

**Tumor designation**	**Pathologic Histology**	**Subtype**	**Primary/ recurrence**
Patient #1	Glioblastoma	PN	Primary
Patient #2	Glioblastoma	PN	Primary
Patient #3	Glioblastoma	CL	Primary

Patient and pathological information associated with brain tumor samples is provided. the tumor histopathology of patients as well as TCGA subtype are included. PN, proneural; CL, classical
.
